# Longitudinal trajectory of vascular age indices and cardiovascular risk factors: a repeated-measures analysis

**DOI:** 10.1038/s41598-023-32443-5

**Published:** 2023-04-03

**Authors:** Daiki Watanabe, Yuko Gando, Haruka Murakami, Hiroshi Kawano, Kenta Yamamoto, Akie Morishita, Nobuyuki Miyatake, Motohiko Miyachi

**Affiliations:** 1 Faculty of Sport Sciences, Waseda University, 2-579-15 Mikajima, Tokorozawa-City, Saitama 359-1192 Japan; 2 Department of Physical Activity Research, National Institute of Health and Nutrition, National Institutes of Biomedical Innovation, Health and Nutrition, 1-23-1 Toyama, Shinjuku-Ku, Tokyo 162-8636 Japan; 3 Faculty of Sport Science, Surugadai University, 698 Azu, Hanno-City, Saitama 357-8555 Japan; 4 Faculty of Sport and Health Science, Ritsumeikan University, 1-1-1 Noji-Higashi, Kusatsu-City, Shiga 525-8577 Japan; 5 Faculty of Letters, Kokushikan University, 4-28-1 Setagaya, Setagaya-Ku, Tokyo 154-8515 Japan; 6 Faculty of Pharmaceutical Sciences, Teikyo Heisei University, 4-21-2 Nakano, Nakano-Ku, Tokyo 164-8530 Japan; 7 Okayama Southern Institute of Health, Okayama Health Foundation, 408-1 Hirata, Okayama-City, Okayama 700-0952 Japan; 8 Faculty of Medicine, Kagawa University, 1750-1 Ikenobe, Miki-Cho, Kita-Gun, Kagawa 761-0793 Japan

**Keywords:** Cardiovascular biology, Ageing, Atherosclerosis, Hypertension, Cardiology, Risk factors

## Abstract

This study aimed to identify the modifiable cardiovascular risk factors associated with longitudinal changes, which are nine functional and structural biological vascular aging indicators (BVAIs), to propose an effective method to prevent biological vascular aging. We conducted a longitudinal study of 697 adults (a maximum of 3636 BVAI measurements) who were, at baseline, aged between 26 and 85 years and whose BVAIs were measured at least twice between 2007 and 2018. The nine BVAIs were measured using vascular testing and an ultrasound device. Covariates were assessed using validated questionnaires and devices. During the mean follow-up period of 6.7 years, the average number of BVAI measurements ranged from 4.3 to 5.3. The longitudinal analysis showed a moderate positive correlation between the common carotid intima-media thickness (IMT) and chronological age in both men (r = 0.53) and women (r = 0.54). In the multivariate analysis, BVAIs were associated with factors such as age, sex, residential area, smoking status, blood clinical chemistry test levels, number of comorbidities, physical fitness, body mass, physical activity, and dietary intake. The IMT is the most useful BVAI. Our findings suggest that modifiable cardiovascular risk factors are associated with longitudinal changes in BVAI as represented by IMT.

## Introduction

Although aging is a major risk factor for cardiovascular disease (CVD)^1^, there are inter-individual differences in CVD onset and the risk for cardiac mortality^2^. There are two types of aging: chronological aging (i.e., time elapsed from birth) and biological aging (i.e., deterioration in tissue and biological function)^3^. Biologically speaking, not everyone ages at the same rate. The biological age of centenarians progresses slower than the chronological age (CA), while the reverse is true for individuals with CVD^3^. Few centenarians have obvious risk factors for CVD^3,4^. Therefore, delay in biological vascular aging is essential for health and longevity. The proportion of adults aged ≥ 65 years is expected to increase from 12 to 22% over the next 30 years in the United States^5^. Additionally, the proportion of the world's population aged ≥ 60 years will nearly double from 12 to 22% between 2015 and 2050. Therefore, it is important to study biological vascular aging to prevent the onset and progression of CVD^6^.

Biological vascular aging indicators (BVAIs) consist mainly of two components, that is, the (1) molecular and cellular component and (2) functional and structural component^3^. Molecular and cellular BVAIs, including the length of the telomere^7^, could reflect various aspects of the aging process, and their trajectories may not be specific to vascular aging^8^. Functional and structural BVAIs include arterial stiffness, blood pressure, endothelial dysfunction, and intima-media thickening^3^. All of these are known risk factors for adverse cardiovascular outcomes and eventual cardiac mortality^9–13^. Given that the longitudinal within-person trajectories of some BVAIs are associated with each other^14^, a comprehensive study is needed to identify more reliable, precise, and accurate BVAIs. Therefore, since CVD is associated with the trajectories of arterial function and phenotypes, it is important to study the arterial biological aging of individuals^3,15^.

Lifestyle factors account for 64.5% of the population-attributable risk fraction for CVD^16^. For example, cigarette smoking^3,17^, physical inactivity^3,18^, and poor diet^3,19^ are all associated with vascular aging. However, these studies have limited their focus on performing baseline measurements of exposure variables at the beginning of the study. Therefore, there is a need to provide insight into individual- and group-level changes in the trajectories of individual BVAIs^20^. To the best of our knowledge, no studies have performed adequate longitudinal research to define the trajectories of different BVAIs.

Through a repeated-measures analysis of BVAI measurements obtained from individuals across a wide age range, this longitudinal study aimed to identify modifiable cardiovascular risk factors associated with longitudinal changes in vascular age indices to propose an effective method to prevent biological vascular aging. We hypothesized that some BVAIs exhibit parallel changes with increasing CA, which could determine modifiable factors associated with vascular aging.

## Methods

### Study design and setting

The current research was conducted as part of a larger prospective cohort study conducted and managed by the National Institute of Health and Nutrition (NIHN) since 2007. This was a multisite cohort study conducted among healthy individuals in Tokyo, the capital of Japan, and Okayama Prefecture, a rural region in Japan, to provide the knowledge to prevent lifestyle-related diseases. Individuals with terminal diseases were excluded. The research details have been described elsewhere^21–23^. A total of 760 individuals (504 in Tokyo, 256 in Okayama Prefecture) participated in this study between 2007 and 2018 (Table S1). These participants were recruited during specific health checkups conducted by the NIHN or the Okayama Southern Institute of Health.

This study was conducted according to the guidelines laid down in the 1964 Declaration of Helsinki and all procedures involving research study participants were approved by the Research Ethics Committee of the National Institutes of Biomedical Innovation, Health and Nutrition (approval no: kenei102-01). Written informed consent was obtained from all participants. In reporting this study, we have followed the Strengthening the Reporting of Observational Studies in Epidemiology guidelines^24^.

### Study sample

From the group that completed the baseline survey (*n* = 760), we excluded those participants who lacked follow-up data (*n* = 60), those for whom age and sex data were missing (*n* = 1), and participants for whom BVAI assessments were not performed (*n* = 2). The sample included 697 Japanese adults aged 26–85 years who completed the baseline examination as well as at least two follow-up assessments of BVAIs and lifestyle risk factors. The investigations were conducted annually using the same survey methodology and content, and the participants were followed up for a maximum of 12 years. Data for the following three samples were used (for sensitivity analysis). First, we performed an analysis in a sample of 690 individuals (a maximum of 3636 measurements) for whom all BVAI data were available. This sample was termed the full analysis set (FAS). Second, we included a sample of 678 individuals (2943 measurements) for whom complete data on all nine BVAIs were obtained through in-person testing. This sample was called the BVAI complete case (BCC). Third, we included 648 individuals (2633 measurements) for whom complete data on all BVAI and lifestyle risk factors could be assessed. This sample was called the complete case (CC).

### Assessment of biological vascular aging indicators

To assess functional and structural BVAIs, systolic blood pressure (SBP), ankle-brachial index (ABI), heart rate (HR), common carotid diastolic diameter (DD), carotid artery mean blood velocity (MBV), blood flow (BF), common carotid intima-media thickness (IMT), carotid-femoral pulse wave velocity (PWV), and vascular aging index (VI) were assessed in the morning after an overnight fasting period of 10 h or more. Details of the assessment methods have been described^21,25^. SBP, ABI, HR, and PWV were measured noninvasively using a vascular testing device (Model BP203RPE II, from PWV/ABI; OMRON Colin Medical Instruments, Tokyo, Japan). While the participants were at rest in the supine position, cuffs were placed on both arms and ankles, electrocardiogram electrodes on both wrists, a cardiac sound sensor on the left sternal border, and a tonometer on the common carotid and femoral artery. A multi-element tonometry sensor (CAP-350 and FAP-350; Colin Medical Technology, Komaki, Japan) was pressed perpendicularly against the wall of the carotid artery and the femoral artery to simultaneously record pulse waveforms of the common carotid and femoral arteries to calculate the carotid-femoral PWV.

The DD, IMT, and MBV were measured using ultrasound devices (Vivid i; GE Medical Systems, USA, and model 180 Plus; Sonosite, USA). While the participants were at rest in the supine position, an ultrasound device with a high-frequency linear array probe at 10 MHz was used to image the longitudinal common carotid artery in B-mode, and the images were recorded as a video. Longitudinal images of the common carotid artery were analyzed using image analysis software (Image J, National Institutes of Health, USA). The mean DD and IMT was calculated from the images using five frames at showing the end diastolic diameter of the left ventricle per cardiac cycle. DD was defined as the distance between near and far lumen-intima interfaces. IMT was defined as the distance between the lumen-intima interface and the medial-adventitial interface^26^. No participants had an IMT ≥ 1.5 mm which is defined as grade 1 plaque^27^. The MBV in the common carotid artery was measured using the above ultrasound device and Doppler ultrasonography. The BF^28^ and VI^10^ of the carotid artery were calculated using the equations from previous studies as follows: BF (mL/min) = MBV (cm/s) × π × DD^2^ (cm^2^) × 60; VI = log_*e*_(1.09) × 10IMT (mm) + log_*e*_(1.14) × PWV (cm/s).

### Assessment of covariates

In this study, previously reported factors associated with BVAIs were analyzed in a comprehensive manner^3^. Participants wore light clothing and their body weight was measured using a digital scale (BC-600, TANITA Corp., Tokyo, Japan). Body mass index (BMI) was calculated as weight divided by height squared (kg/m^2^). We calculated the waist/hip ratio as the abdominal obesity index by measuring the waist (at the height of the navel) and the hip circumference (at the largest bulge perpendicular to the long axis of the trunk). The trunk flexibility was measured using a sit-and-reach digital instrument (T.K.K.5112; Takei Scientific Instruments Co. Ltd., Niigata, Japan). Regarding leg strength, unidirectional lower limb extension strength was measured using a multi-joint leg extension apparatus (Anaeropress 3500; Combi Co., Tokyo, Japan). The grip strength was measured using a Smedley hand dynamometer (Grip-D TKK5101, Takei Scientific Instruments, Niigata, Japan). Measurements were performed twice on each hand; the highest value for each hand was used for analysis. The following biochemical parameters were measured: red blood cell count, white blood cell count, platelet count, hemoglobin, high-density lipoprotein cholesterol, low-density lipoprotein cholesterol (LDL-C), triglycerides, hemoglobin A1c (HbA1c), homeostasis model assessment of insulin resistance (HOMA-IR), aspartate aminotransferase (AST), alanine aminotransferase (ALT), and γ-glutamyl transpeptidase (γ-GTP). As an objective measure of physical activity, step counts were measured using a previously validated triaxial accelerometer (Actimarker EW4800, Panasonic, Osaka, Japan)^29^. The n-3/n-6 fatty acid ratio and the intake of saturated fatty acids (SFA), alcohol, salt, sugar, meat, fruits and vegetables (FV), and pulses were assessed using a dietary questionnaire that was validated against the dietary record method^30,31^. We calculated the food and nutrient intake per 1000 kcal using the density method to adjust for the energy intake^32^. Questionnaires were administered to assess demographic information, smoking status, family history of heart disease, presence of comorbidities, and sleep status. Comorbidities were classified as a comorbidity score based on the total number of comorbidities, out of 10, that were present in the participants—hypertension, dyslipidemia, diabetes, ischemic heart disease, other heart diseases, cerebrovascular disease, renal failure, cancer, osteoporosis, and mood disorders^22,23^.

### Statistical analysis

Regarding participant characteristics, continuous variables are presented as mean (standard deviation), while categorical variables are presented as number (percentage). The missing covariate values (see Supplementary Material) were supplemented with five datasets using multivariate imputation by chained equation in R Statistical Software^33^. All missing values were treated as missing at random.

We identified the longitudinal trajectories of BVAIs using latent growth curve models (LGCMs) and latent class growth models from repeated BVAI measurement data (FAS dataset). These analyses were performed using the STATA macro TRAJ^34^. After stratifying by sex, the overall mean trajectory of BVAIs was estimated using LGCMs (cubic splines).

The results of the cross-sectional and repeated longitudinal analyses were compared using the BCC dataset to identify the between- and within-person trajectory of BVAIs^35^. This analysis was stratified by age group (≤ 39 years, 40–49 years, 50–59 years, 60–69 years, and ≥ 70 years) assuming heterogenous age-related trajectories of BVAIs^36^. The CA-related trajectories of nine BVAIs were assessed using univariate panel data regression analysis. The results are presented as regression coefficients and 95% confidence intervals by changes per year of CA. Furthermore, to assess the correlation coefficients of cross-sectional and longitudinal repeated analyses between CA and BVAIs^36^, a longitudinal analysis was performed using Repeated Measures Correlation (rmcorr) by R Statistical Software^37^, and a cross-sectional analysis was performed using the Pearson correlation coefficient.

To assess the parallel changes in factors associated with changes over time in the longitudinal trajectories of nine BVAIs, we performed a multivariate regression analysis of the random effect panel data (using baseline covariate data and longitudinal trajectories)^22,23^. The variables mentioned above were used as covariates for the multivariate analysis model. Variables with a variance inflation factor (VIF) ≤ 10 were used in the model to avoid multicollinearity^38^. If the VIF was > 10, variables with the highest predicted value were maintained in the model (Table S2). Results are presented as regression coefficients and 95% confidence intervals per unit of the relevant variable. Sensitivity analysis was similarly performed using three datasets (FAS, BCC, and CC).

Statistical significance was established at a two-tailed *P* < 0.05 (z score, ≤  − 1.96 or ≥ 1.96). All analyses were performed using STATA MP, version 15.0 (StataCorp LP, College Station, TX, USA) or R software 3.4.3 (R Core Team, Vienna, Austria).

## Results

Of the 697 participants, 69.6% were women and 33.3% lived in local areas (Table 1). The mean age and BMI were 51.4 years and 22.5 kg/m^2^, respectively. On average, the levels of BVAIs were 120 mmHg for SBP, 1.15 for ABI, 63 bpm for HR, 6.1 mm for DD, 28.3 cm/s for MBV, 515 mL/min for BF, 0.62 mm for IMT, 836 cm/s for PWV, and 32.9 for VI. The baseline characteristics of the individuals in the BCC and CC datasets were similar to those of the FAS dataset (Table S3).

**Table 1:** Number and characteristics of baseline (first available) measurements with information on biological vascular aging indicators.

	Baseline and follow-up measurements in individuals											
	FAS				BVAI complete case				All complete case			
	*n*	MT	Distribution		*n*	MT	Distribution		*n*	MT	Distribution	
Age [years]	697	4880	52.5	(11.5)	678	2936	52.6	(11.5)	648	2633	52.7	(11.5)
Women [*n* (%)]	697	4880	485	(69.6)	678	2936	473	(69.8)	648	2633	451	(69.6)
Local area [*n* (%)]	697	4880	232	(33.3)	678	2936	219	(32.3)	648	2633	199	(30.7)
Body mass index [kg/m^2^]	697	4181	22.5	(2.9)	678	2936	22.5	(2.9)	648	2633	22.5	(2.9)
Comorbidity score, 0 score	697	4676	529	(75.9)	678	2914	516	(76.1)	648	2633	492	(75.9)
Smoker [n (%)]	697	3796	198	(28.4)	678	2781	188	(27.7)	648	2633	182	(28.1)
Alcohol drinker [*n* (%)]	697	4135	514	(73.7)	678	2900	502	(74.3)	648	2633	482	(74.5)
BVAIs												
SBP [mmHg]	690	3636	120	(16)	678	2936	120	(16)	648	2633	120	(16)
ABI	686	3341	1.15	(0.08)	678	2936	1.15	(0.07)	648	2633	1.15	(0.07)
HR [bpm]	686	3341	63	(12)	678	2936	63	(11)	648	2633	62	(11)
DD [mm]	681	3003	6.1	(0.7)	678	2936	6.1	(0.7)	648	2633	6.1	(0.7)
MBV [cm/s]	686	3161	28.3	(7.1)	678	2936	28.3	(7.2)	648	2633	28.4	(7.2)
BF [mL/min]	681	2995	515	(141)	678	2936	515	(141)	648	2633	517	(141)
IMT [mm]	681	3002	0.62	(0.11)	678	2936	0.62	(0.11)	648	2633	0.62	(0.11)
PWV [cm/s]	685	3279	836	(167)	678	2936	835	(166)	648	2633	835	(166)
VAI	678	2943	32.9	(4.9)	678	2936	32.9	(4.9)	648	2633	32.9	(4.9)
Follow-up period [years]	697		6.7	(2.5)	678		6.7	(2.5)	648		6.8	(2.4)

The variables are presented as mean (standard deviation) or number of cases (percentage). FAS refers to using all acquired data. A total of 697 individuals were included in the data of participants characteristics, including age (4880 measurements), while a maximum of 690 individuals were included in the BVAI data (3636 measurements). BCC refers to using the data on all nine BVAIs acquired at the same in-person testing (no missing data exclusively for BVAIs). CC refers to using data obtained at the same in-person testing for all covariates included in the multivariate analysis (no missing data for any covariates).

*ABI* ankle-brachial index, *BCC* BVAI complete case, *BF* blood flow, *BVAI* biological vascular aging indicator, *CC* complete case, *DD* common-carotid diastolic diameter, *FAS* full analysis set, *HR* heart rate, *IMT* common-carotid intima-media thickness, *MBV* mean blood velocity, *MT* measurement times, *PWV* carotid-femoral pulse wave velocity, *SBP* systolic blood pressure, *VI* vascular aging index.

**Figure 1:**
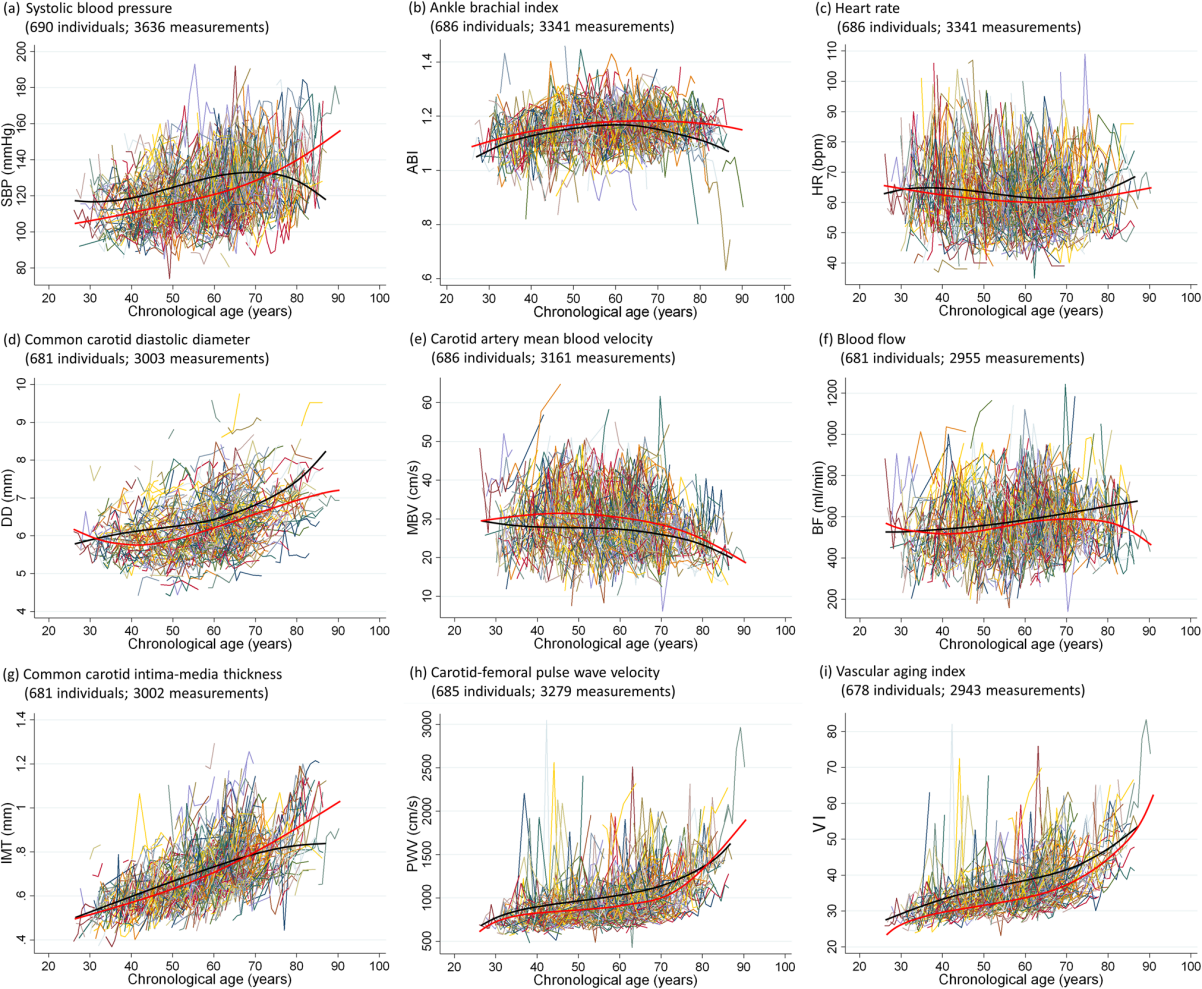
Longitudinal trajectories of biological vascular aging indicators (BVAIs) in the full analysis set (at least two BVAIs). A total of 678 individuals were included in the full set analysis of BVAIs of the vascular aging index (number of measurements: 2943), while a total of 690 individuals were included in the full analysis set of BVAIs of systolic blood pressure (number of measurements: 3636). The general mean trajectories of BVAIs, stratified into men (black line) and women (red line), were estimated using models of latent growth curves. *ABI* ankle-brachial index, *BF* blood flow, *DD* common carotid diastolic diameter, *HR* heart rate, *IMT* common carotid intima-media thickness, *MBV* carotid artery mean blood velocity, *PWV* carotid-femoral pulse wave velocity, *SBP* systolic blood pressure, *VI* vascular aging index.

The mean number of BVAI measurements ranged from 4.3 (VI) to 5.3 (SBP). The mean follow-up period was 6.7 years. The longitudinal trajectories of nine BVAIs using cubic spline models by sex are shown in Fig. 1. We found sex differences in CA-related BVAI trajectories (Fig. S1); young women showed slower vascular aging than young men, while older adults showed smaller sex differences. The mean SBP (for women), DD, IMT, PWV, and VI increased, while MBV and BF (for women) decreased with CA. The rate of change in BVAIs tended to accelerate in women in their 60 s and men in their 70 s. Other variables had the lowest or no curvature. Additionally, we identified two to four discrete patterns of the trajectory groups of the BVAIs. The mean trajectory of DD, IMT, PWV, and VI tended to have larger differences between the trajectory groups in young adults than they did in older adults (Fig. S2).

**Figure 2:**
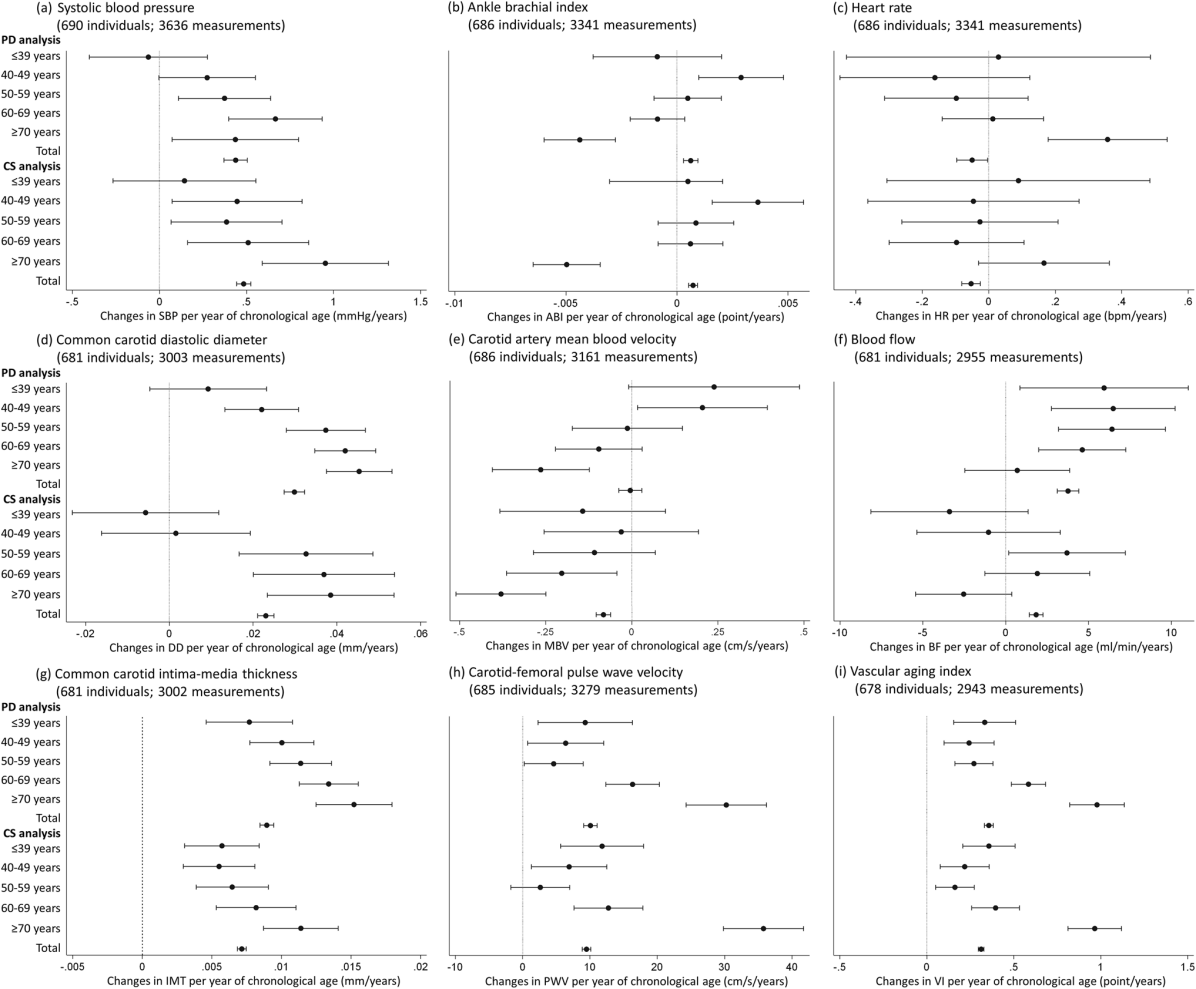
Comparison of cross-sectional and panel data analyses by biological vascular aging indicator effect per year of chronological age. The values are presented as regression coefficients and 95% confidence intervals. *ABI* ankle-brachial index, *BF* blood flow, *CS* cross-sectional, *DD* common carotid diastolic diameter, *HR* heart rate, *IMT* common carotid intima-media thickness, *MBV* carotid artery mean blood velocity, *PD* panel data, *PWV* carotid-femoral pulse wave velocity, *SBP* systolic blood pressure, *VI* vascular aging index.

**Figure 3:**
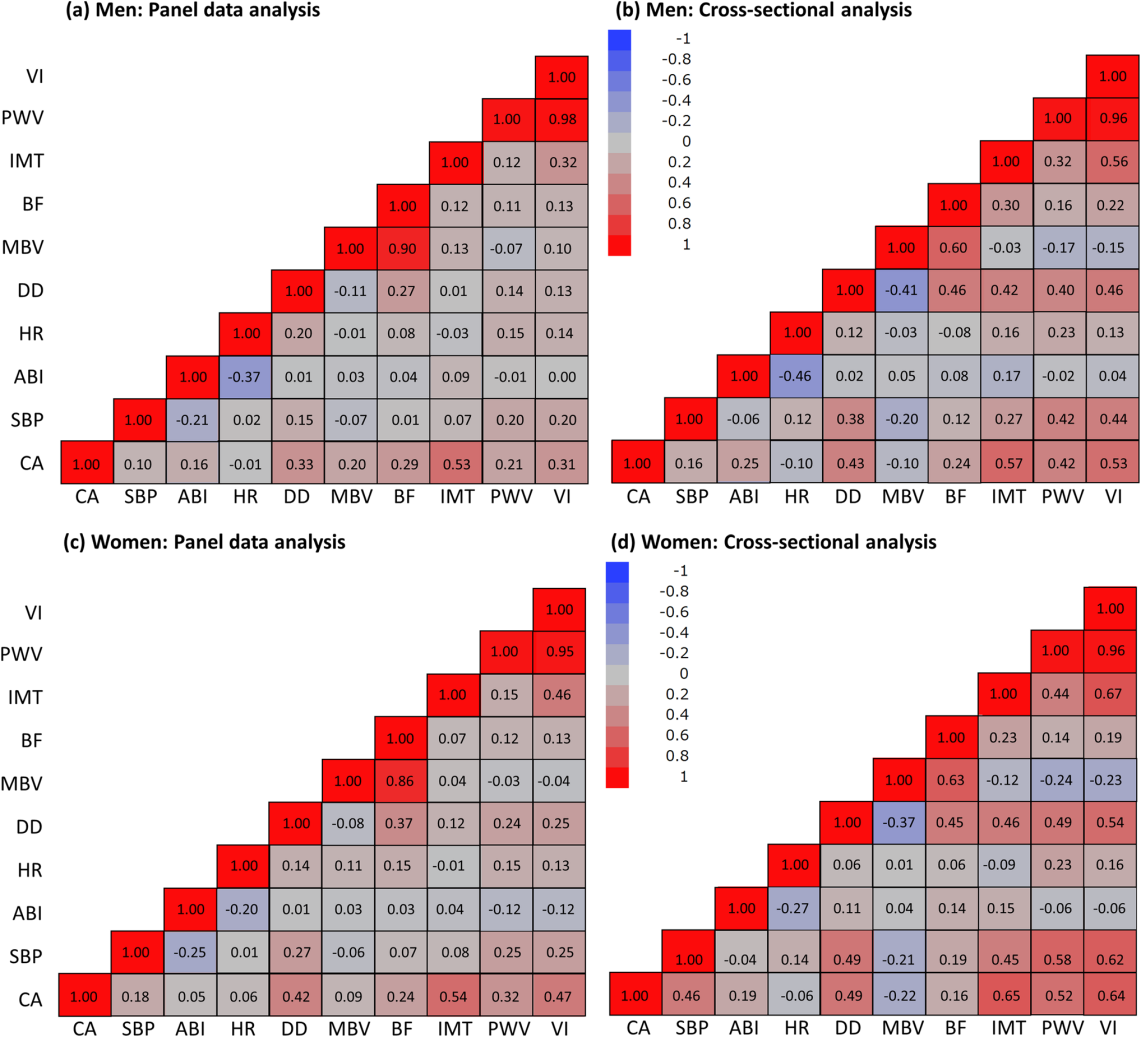
Comparison of interindividual correlations between biological vascular aging indicators and chronological age in 678 individuals. The complete case dataset of the biological vascular aging indicators (678 individuals, 2936 complete measurements) was included in the analysis of within-person (a, men; c, women) and between-person (b, men; d, women) correlations. The red and blue tiles show positive and negative correlations, respectively. The color density represents the degree of correlation, where a higher density represents a stronger correlation. *ABI* ankle-brachial index, *BF* blood flow, *CA* chronological age, *DD* common carotid diastolic diameter, *HR* heart rate, *IMT* common carotid intima-media thickness, *MBV* carotid artery mean blood velocity, *PWV* carotid-femoral pulse wave velocity, *SBP* systolic blood pressure, *VI* vascular aging index.

The effects of BVAIs per CA year were compared in cross-sectional and longitudinal within-person analyses using the BCC dataset (Fig. 2). The results per age group showed different trends and estimated values. The results of the cross-sectional and within-person longitudinal correlations between nine BVAIs and CA are shown in Fig. 3. Compared to the longitudinal correlation analysis, cross-sectional correlations were stronger in men and women. The longitudinal correlation analysis showed a moderate positive correlation between IMT and CA in men (*r* = 0.53) and women (*r* = 0.54). The cross-sectional analysis showed similar results. Only women showed a significant correlation between VI and age in the longitudinal (*r* = 0.47) and cross-sectional (*r* = 0.64) analyses. There were no significant correlations between other variables except those used in the calculation. The fraction attributable to between-person variance in the change in BVAIs ranged from 52% (ABI) to 75% (DD) (Table S4), showing similar results when stratified by sex (Tables S5, S6).

**Figure 4:**
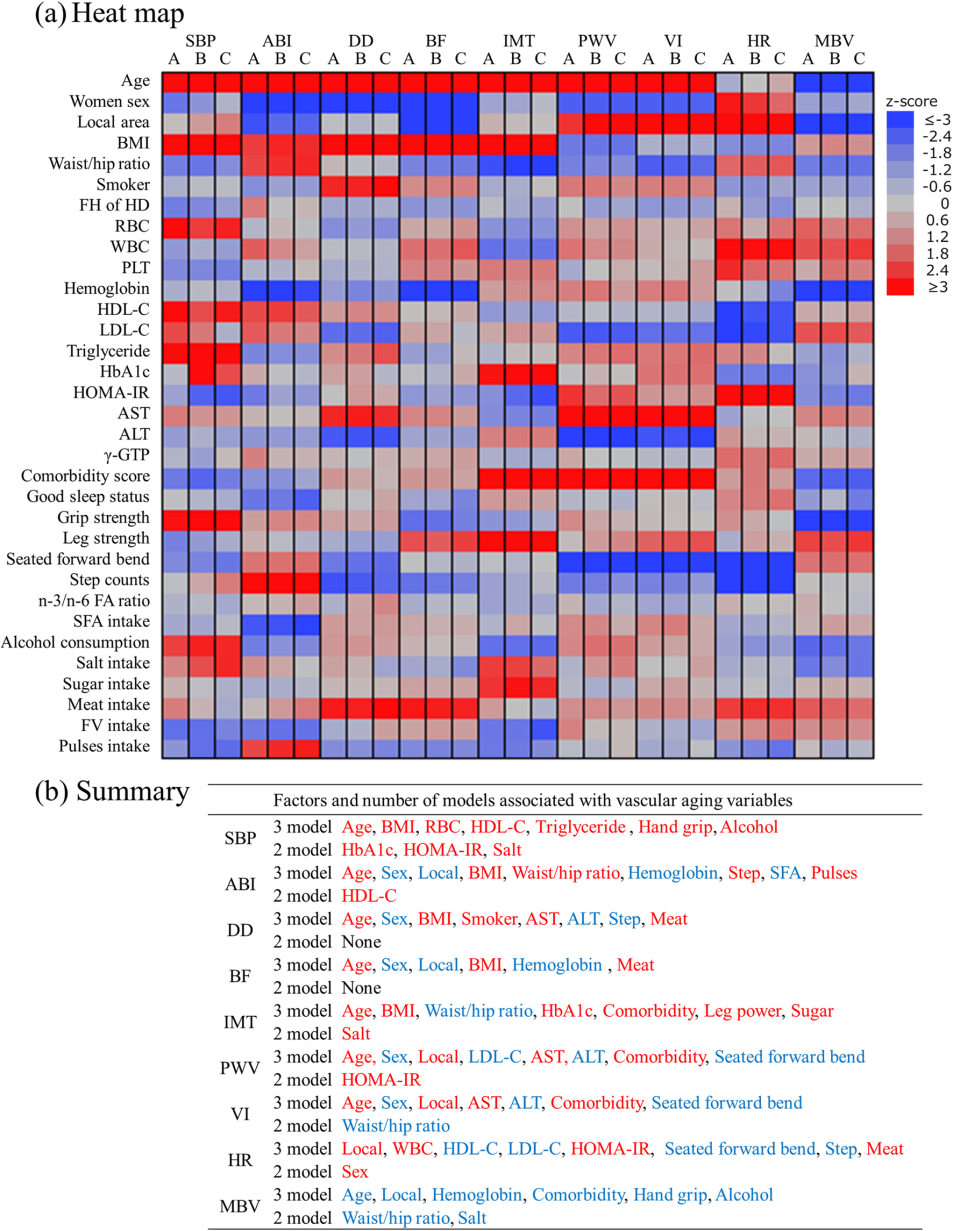
Factors associated with biological vascular aging indicators (BVAIs) identified using a multivariate panel data analysis using the three models. (**a**) Heatmap of the factors associated with BVAIs; the results of the analysis are presented as z-scores. We used ggplot2 package in R software 3.4.3 (R Core Team, Vienna, Austria) to generate heatmaps. Red and blue tiles represent positive and negative correlations, respectively. The color density represents the magnitude of the z-scores, with a higher density representing a higher z-score. The analysis was performed using the following three models, considering a sensitivity analysis: A, complete analysis set; B, complete BVAI case set; C, complete case set. (**b**) Summary of the factors related to BVAIs; red and blue variables represent positive and negative correlations. Sex, area, and FH of HD were time-stable variables, while the other covariates were time-varying variables. Regarding the column “3 models” and “2 models”: 3 and 2, respectively, indicate the number of models (of the three models) that show significant associations; “3 model” suggests the results are robust. The detailed results of all analyses are shown in the supplementary material. *ABI* ankle-brachial index, *ALT* alanine aminotransferase, *AST* aspartate aminotransferase, *BF* blood flow, *BMI* body mass index; *DD* common carotid diastolic diameter, *FA* fatty acid, *FH* family history, *FV* fruits and vegetables, *HbA1c* hemoglobin A1c, *HD* heart disease, *HDL-C* high-density lipoprotein cholesterol, *HOMA-IR* homeostasis model assessment of insulin resistance, *HR* heart rate, *IMT* common carotid intima-media thickness, *LDL-C* low-density lipoprotein cholesterol, *MBV* carotid artery mean blood velocity, *PLT* platelet, *PWV* carotid-femoral pulse wave velocity, *RBC* red blood cells, *SBP* systolic blood pressure, *SFA* saturated fatty acid, *VI* vascular aging index, *WBC* white blood cell, *γ-GTP* γ-glutamyl transpeptidase.

To assess parallel changes in factors associated with changes over time in the longitudinal trajectories of nine BVAIs, we performed multivariate panel data analysis using three models (Fig. 4, Tables S7–S157). BVAIs were significantly associated with age, sex, regional location, smoking status, blood clinical chemistry test levels, number of morbidities, physical fitness (grip strength, leg strength, and body flexibility), physique, physical activity, and dietary intake. Three models for sensitivity analysis showed consistent associations for many variables.

## Discussion

We found sex differences in the longitudinal trajectories of nine functional and structural BVAIs. The longitudinal and cross-sectional correlations between BVAIs and CA differed. However, both analyses showed a moderate correlation between IMT and CA. We also found that BVAIs change in parallel with alterations in modifiable lifestyle factors associated with CVD risk. To our knowledge, this is the first study to identify better BVAIs and factors changing in parallel with BVAI trajectories. The strength of this study lies in the high participation rate of the same individuals in whom we assessed the nine BVAIs. A more accurate evaluation was possible owing to the longitudinal assessment of covariates using a validated method, which led to the comprehensive determination of factors associated with BVAIs by minimizing bias due to between-person variance.

The cross-sectional and longitudinal analyses used in the present study showed a significant positive correlation between IMT and CA in men and women, consistent with previous studies^14^. However, no studies performed cross-sectional and longitudinal analyses of functional and structural BVAIs using the same samples. The findings of the cross-sectional study are limited to elucidating changes at the group level. Therefore, it is impossible to determine age-related changes in individual-specific BVAIs^20^. Furthermore, cross-sectional studies may not be suitable given that they are affected by confounding factors and cohort effects^20^. In this study, the interrelationships between BVAIs and CA were stronger in the cross-sectional than longitudinal analysis. Variables for which the between-person correlation coefficient (cross-sectional analysis) is significantly higher than the within-person correlation coefficient (longitudinal analysis) may reflect individual differences. Therefore, these variables can bias the assessment of BVAIs in individuals or reduce the association. It is necessary to associate CA with both cross-sectional and individual longitudinal analyses as a criterion for biomarkers of aging^39^. Our findings indicated that IMT had the strongest relationship with CA in the longitudinal analysis. The relationships may be considered irreversible because IMT is a morphological and anatomical index unlike BP and PWV which are functional and physiological indices^3^. The length of telomeres as a typical aging marker is also irreversible because it cannot be restored in humans^3,7^. Our results showed that IMT had a linear relationship with CA compared with other BVAIs, suggesting that IMT can be used as a BVAI in individuals across a wide age range (26–90 years). Moreover, previous longitudinal studies showed a significant and independent association between IMT and adverse cardiovascular events^10,13^. In contrast, BVAIs, which have a curved relationship with CA, may partly explain the mechanism of vascular events that occur in old age. Therefore, the IMT assessment using carotid artery ultrasound as a noninvasive and inexpensive method is a more useful BVAI assessment in both men and women.

Our study found that young women showed slower vascular aging than did young men, while older adults showed smaller sex differences. We also showed that the rate of change in BVAIs tended to accelerate in women in their 60 s and men in their 70 s. A previous study also reported sex differences in functional biological aging and its rapid changes around the age of 70 years^36^; these findings support our results. Many women develop CVD a few years later than men^40^. This acceleration may be due to menopause in middle-aged and older women. Menopause was not identified as a significant risk factor for incident CVD in previous guidelines^41^; however, recent statements have indicated otherwise^42^. Natural menopause occurs in approximately 75% of women in their 50s^43^. The age-related increase in IMT and adventitial diameter occurs more rapidly during postmenopause than premenopause^44^. Our study showed rapid vascular aging in women in their 60 s, most of whom reached menopause. Previous data support our findings. This study identified two to four discrete groups of BVAI trajectories, demonstrating the unlikeliness of everyone having similar BVAI trajectories. Because we were unable to obtain the menopausal status of women, sex differences in hormones, genetic factors, and menopause should be investigated in more detail^45^. Therefore, efforts related to assessing BVAIs and vascular protection in middle-aged adults are needed to prevent the increased risk of CVD events in older adults.

In this study, we showed a significant association between BVAIs and modifiable lifestyle risk factors. Similar results were found in multiple-sensitivity analyses. Given that human aging is caused by a combination of genetic and environmental factors, the speed of aging is heterogeneous^46^. Therefore, the main pathophysiological mechanisms of aging may not be accounted for by a single factor, and it is necessary to verify the various factors associated with biological vascular aging. The American Heart Association recommends “Life’s Simple 7” to maintain cardiac health^6^. Those who follow the recommendations have a lower risk of developing CVD^16^. In the current study, five of six items used as dependent variables (smoking, diet, step count, BMI, and HbA1c) were associated with the trajectories of BVAIs. Furthermore, we found an association between increased muscle strength (e.g., grip and leg strength) and increased SBP or IMT, and between increased trunk flexibility and decreased PWV, VI, and HR. Our previous intervention^25^ and prospective^21^ studies reported significant associations between these variables and vascular compliance. Therefore, these variables are also important as modifiable factors of BVAIs. We have demonstrated the benefits of an increase in ALT or LDL-C in some BVAIs. However, these results are inconsistent with those of previous studies^47,48^ and therefore require further investigation. Although our results suggest that improving lifestyle and other modifiable factors prevent vascular aging, detailed mechanisms and causal relationships should be elucidated by basic and interventional research. Few centenarians have explicit CVD risk factors^4^. Our findings may be useful in developing health guidance programs that are used in the clinical and public health fields to prevent vascular aging and promote healthy longevity.

There are some methodological limitations. First, participants were not selected by random sampling and may have had greater health awareness than the general population does. Healthy individuals included may have experienced fewer changes compared to the general population. In addition, the observation period in our study was relatively short. Vascular calcification, included in BVAIs, were not examined in this study. Because our study excluded patients with severe CVD, participants with large, well-defined, pathological carotid plaques were not included. Consequently, as shown in the IMT panel of Fig. 1, none of the participants had an IMT ≥ 1.5 mm which is defined as grade 1 plaque in the American Society of Echocardiography guidelines^27^. The longitudinal age-related trajectories of these variables must be assessed in a cohort study that includes participants with a higher atherosclerotic burden. Therefore, the results of this study need to be reexamined in a long-term follow-up of young groups with fewer vascular aging compared to groups with more advanced vascular aging. Second, to avoid excluding potentially interesting associations, the significance level was not adjusted for^49^. However, calculating *P*-values for multiple tests can lead to multiple comparisons. Therefore, the present results need to be verified by further and more detailed studies. Furthermore, the small sample size stratified by sex may have affected the results. However, using previously reported equation^22,23,50^, the accuracy and precision of our BVAIs were adequate based on the estimated sample sizes and the study period used in our study (Tables S4–S6). Third, this study could not measure biomarkers of biological age. It is needed to measure a more specific indicator of biological aging, including the length of the telomere, because aging itself is a natural phenomenon, but there is a great difference between biological and chronological aging. However, the mortality risk is associated with the Frailty Index, assessed by the accumulated deficit of functional phenotype but not telomere length^36^, and it remains unknown whether telomere attrition promotes CVD^3^. Therefore, biological age is a construct built not only on molecular and cellular but also on functional aspects of an individual’s health status. Finally, the present results may have been affected by unmeasured confounders. Although we examined economic and medication status, we were unable to use these variables as covariates because nearly all data contained missing values. Often, in people with disease whose BVAIs are normalized by pharmacotherapy, the effects of longitudinal trajectories of BVAIs are underestimated. These limitations prohibit the generalization of our findings. Therefore, a well-designed longitudinal cohort study with larger randomized samples is necessary to further determine the parallel changes in factors associated with temporal changes in BVAI.

This study identified modifiable lifestyle factors associated with longitudinal changes in BVAI trajectories. Moreover, it demonstrated the importance of further investigation to identify groups that may benefit most from the amelioration of modifiable risk factors for premature vascular aging. Considering that humans cannot avoid chronological aging, our findings provide new insights into individual differences in biological vascular aging and demonstrate the importance of addressing lifestyle-related risk factors in young, middle-aged, and older individuals to prevent the development of CVD later in life.

## Conclusion

The IMT was the most useful BVAI in both sexes. We showed rapid changes in BVAIs in women in their 60 s and men in their 70 s, and we identified modifiable lifestyle risk factors such as obesity, metabolic disorders, physical inactivity, and poor dietary habits associated with longitudinal changes in BVAI trajectories. There is an urgent need to identify an effective lifestyle intervention that promotes the modification of risk factors associated with premature vascular aging. In this regard, our findings may provide useful information on effective preventive interventions for biological vascular aging.

## Supplementary Information

Supplementary Information.

## Data Availability

The datasets used and analyzed during the current study are available from the corresponding author on reasonable request (cardiovascular0327@mac.com).

## Abbreviations

ABI: Ankle-brachial index
AST: Aspartate aminotransferase
ALT: Alanine aminotransferase
BCC: BVAI complete case
BF: Blood flow
BMI: Body mass index
BVAI: Biological vascular aging indicator
CA: Chronological age
CC: Complete case
CVD: Cardiovascular disease
DD: Common carotid diastolic diameter
FAS: Full analysis set
FV: Fruits and vegetables
γ-GTP: γ-Glutamyl transpeptidase
HbA1c: Hemoglobin A1c
HOMA-IR: Homeostasis model assessment of insulin resistance
HR: Heart rate
IMT: Common carotid intima-media thickness
LDL-C: Low-density lipoprotein cholesterol
LGCM: Latent growth curve model
MBV: Carotid artery mean blood velocity
NIHN: National Institute of Health and Nutrition
PWV: Carotid-femoral pulse wave velocity
SBP: Systolic blood pressure
SFA: Saturated fatty acid
VI: Vascular aging index
VIF: Variance inflation factor
