# Supplementary material for: Longitudinal trajectory of vascular age indices and cardiovascular risk factors: a repeated-measures analysis
Source: Sci Rep. 2023 Apr 3;13:5401. doi: 10.1038/s41598-023-32443-5 (PMC10070355; doi:10.1038/s41598-023-32443-5)
Supplement: Supplementary file 1 — Supplementary Information. [file 41598_2023_32443_MOESM1_ESM.docx]

**Supplemental Material**

“Longitudinal trajectory of vascular age indices and cardiovascular risk factors: A repeated-measures analysis”

Authors: Daiki Watanabe, R.D., Ph.D., Yuko Gando, Ph.D., Haruka Murakami, R.D., Ph.D., Hiroshi Kawano, Ph.D., Kenta Yamamoto, Ph.D., Akie Morishita, Nobuyuki Miyatake, M.D., Ph.D., Motohiko Miyachi, Ph.D.

**SUPPLEMENTAL TABLES**

**Supplemental Table 1**. Number of individuals with information on all variables obtained from in-person testing.

**Supplemental Table 2**. Assessment of multicollinearity by vascular aging index for the multivariate panel data analysis.

**Supplemental Table 3.** Number and characteristics of baseline (first available) measurements of individuals with information on all covariates.

**Supplemental Table 4**. Accuracy and precision of BVAIs in all participants.

**Supplemental Table 5**. Accuracy and precision of BVAIs in men.

**Supplemental Table 6**. Accuracy and precision of BVAIs in women.

**Supplemental Table 7.** Multivariate panel data analysis for SBP and vascular aging–related covariates trajectory.

**Supplemental Table 8.** Multivariate panel data analysis for ABI and vascular aging–related covariate trajectory.

**Supplemental Table 9.** Multivariate panel data analysis for HR and vascular aging–related covariate trajectory.

**Supplemental Table 10.** Multivariate panel data analysis for DD and vascular aging–related covariate trajectory.

**Supplemental Table 11.** Multivariate panel data analysis for MBV and vascular aging–related covariate trajectory.

**Supplemental Table 12.** Multivariate panel data analysis for BF and vascular aging–related covariate trajectory.

**Supplemental Table 13.** Multivariate panel data analysis for IMT and vascular aging–related covariate trajectory.

**Supplemental Table 14.** Multivariate panel data analysis for PWV and vascular aging–related covariate trajectory.

**Supplemental Table 15.** Multivariate panel data analysis for VI and vascular aging–related covariate trajectory.

**SUPPLEMENTAL FIGURES**

**Supplemental Figure 1.** Effect of BVAIs per year of chronological age by age and sex-stratified model.

**Supplemental Figure 2.** Trajectory groups by trajectory modeling for nine BVAIs.

**Supplemental Methods**

Among the 690 people [3,636 measurements] (in the full set analysis of SBP, which is the maximum number of people to be analyzed), missing values were supplemented by multiple imputation: BMI (one measurement); waist/hip ratio (319 measurements); smoking status (299 measurements); family history of heart disease (181 measurements); RBC (42 measurements); WBC (74 measurements); PLT (56 measurements); hemoglobin (42 measurements); HDL-C (19 measurements); LDL-C (131 measurements); triglycerides (18 measurements); HbA1c (60 measurements); HOMA-IR (299 measurements); AST (22 measurements); ALT (21 measurements); γ-GTP (19 measurements); comorbidity score (102 measurements); sleep status (304 measurements); hand grip (313 measurements); leg power (314 measurements); seated forward bend (316 measurements); step counts (168 measurements); and dietary intake (43 measurements). Among the 678 people [2,936 measurements] in the BVAI complete case set, missing values were supplemented by multiple imputation: waist/hip ratio (22 measurements); smoking status (155 measurements); family history of heart disease (28 measurements); RBC (five measurements); WBC (five measurements); PLT (10 measurements); hemoglobin (five measurements); HDL-C (four measurements); LDL-C (5 measurements); triglycerides (four measurements); HbA1c (five measurements); HOMA-IR (six measurements); AST (five measurements); ALT (four measurements); γ-GTP (four measurements); comorbidity score (22 measurements); sleep status (158 measurements); hand grip strength (16 measurements); leg power (224 measurements); seated forward bend (19 measurements); step counts (85 measurements); and dietary intake (36 measurements).

The latent class growth models (LCGMs) were estimated using the maximum likelihood method to determine whether the participants could be divided into multiple trajectory groups. The trajectories for the latent growth curve models and LCGMs were interpolated using cubic splines. The Bayesian information criteria and the proportional size of the group (≥5.0%) were used to determine the optimal number of trajectories. Panel data analysis adjusts between-person variance that cannot be controlled by time-series and cross-sectional data alone, and provides a more accurate estimation of the relationship between within-person exposure variables and outcomes by allowing less susceptibility to multicollinearity and influence from confounding factors.

We evaluated the accuracy and precision of the BVAI trajectory estimation from the coefficient of between‐person variance (CV_b_), coefficient of within‐person variance (CV_w_), and within‐person/between‐person variance ratio (VR) by using previously reported equations. The CV_b_ and CV_w_ for BVAIs were calculated using one-way analysis of variance. To evaluate the contribution of CV_b_ and CV_w_ to the BVAI trajectory, we calculated the intra-class correlation coefficients (ICC) using the following equation: ICC = CV_b_ / (CV_w_ + CV_b_). If the ICC is comparatively low, it means that an individual longitudinal panel data analysis is necessary due to the larger CV_w_ of the BVAIs. The accuracy and precision of the BVAI trajectory were calculated using the following equation: 1) the group size (Gs) = 1.96^2^ × [(CV_b_^2^ + CV_w_^2^)/D_0_^2^] required to estimate a group’s “true” mean BVAI within a 95% confidence interval (CI) with a specified % deviation (D_0_), where D_0_ is used as the specified % deviation at 2.5%, 5%, 10%, or 20%; 2) the number of BVAI survey administrations per year (NT_1_) = [*r*^2^/(1 − *r*^2^)] × VR required to obtain a specified correlation coefficient (*r*) between an individual’s measured value and unmeasured usual “true” mean daily step count, where *r* is the specified correlation coefficient (0.85, 0.90, and 0.95) and an index of confidence related to an individual’s classification or ranking within a population. Since each measurement variable is acquired every year in the cohort study, the calculated value is the number of BVAI measurements required for the annual survey; 3) the number of BVAI survey administrations per year (NT_2_) = (1.96 × CV_w_/D_1_)^2^ required to estimate an individual’s “true” mean BVAI within a 95% CI with a specified % deviation (D_1_), where D_1_ is used as the specified % deviation at 5%, 10%, or 20%. In addition, these analyses were performed after stratification by sex.

**Supplemental Results**

Supplemental Tables S3–S6 show the accuracy and precision of the BVAIs. The larger values of the between‐person variance, within‐person variance, and within‐person/between‐person variance ratio imply that a larger population and number of survey days are required for BVAI assessment. The group sizes required to estimate a group’s “true” mean BVAI trajectory within a 95% CI with 2.5% deviation ranged from 19 people (ankle-brachial index) to 343 people (blood flow). The number of BVAI survey administrations per year required to obtain a correlation coefficient (*r*) of 0.90 between an individual’s measured value and “true” unmeasured usual mean BVAI trajectory ranged from once (common carotid diastolic diameter) to 4 times (ankle-brachial index). The number of BVAI survey administrations per year required to estimate an individual’s “true” mean BVAI trajectory within a 95% CI with 5% deviation ranged from once (common carotid diastolic diameter) to 33 times (blood flow). The ICC ranged from 0.52 (ankle-brachial index) to 0.75 (common carotid diastolic diameter). In addition, no great difference by sex was observed in these variances. These results suggest that the accuracy and precision of our BVAI estimations were adequate based on the sample sizes (within 2.5% deviation) and number of survey administrations (“true” correlation 0.9 or more) used in our study.

**Supplemental Table S1.** Number of individuals with information on all variables obtained from in-person testing

|  | In-person testing (*n* = 697) | | | | | | | | | | | | |
| --- | --- | --- | --- | --- | --- | --- | --- | --- | --- | --- | --- | --- | --- |
|  | 1 | 2 | 3 | 5 | 6 | 7 | 8 | 9 | 10 | 11 | 12 | 13 | Total |
| Years of investigation | 2007 | 2008 | 2009 | 2010 | 2011 | 2012 | 2013 | 2014 | 2015 | 2016 | 2017 | 2018 |  |
| SBP | 242 | 368 | 481 | 460 | 347 | 400 | 362 | 283 | 206 | 333 | 169 | 11 | 3662 |
| ABI | 242 | 368 | 481 | 450 | 287 | 336 | 303 | 229 | 174 | 321 | 169 | 11 | 3371 |
| HR | 242 | 368 | 481 | 450 | 287 | 336 | 303 | 229 | 174 | 321 | 169 | 11 | 3371 |
| DD | 240 | 351 | 467 | 425 | 260 | 245 | 214 | 165 | 174 | 321 | 169 | 11 | 3042 |
| MBV | 239 | 350 | 467 | 429 | 270 | 332 | 227 | 200 | 174 | 320 | 169 | 11 | 3188 |
| BF | 237 | 350 | 467 | 425 | 260 | 244 | 211 | 165 | 174 | 320 | 169 | 11 | 3033 |
| MT | 240 | 351 | 467 | 425 | 260 | 245 | 213 | 165 | 174 | 321 | 169 | 11 | 3041 |
| PWV | 214 | 362 | 480 | 447 | 281 | 332 | 299 | 228 | 171 | 317 | 168 | 11 | 3310 |
| VI | 212 | 347 | 466 | 422 | 254 | 242 | 210 | 165 | 171 | 317 | 168 | 11 | 2985 |
| Age | 242 | 369 | 482 | 533 | 480 | 572 | 459 | 468 | 393 | 433 | 411 | 38 | 4880 |
| Sex | 242 | 369 | 482 | 533 | 480 | 572 | 459 | 468 | 393 | 433 | 411 | 38 | 4880 |
| Area | 242 | 369 | 482 | 533 | 480 | 572 | 459 | 468 | 393 | 433 | 411 | 38 | 4880 |
| BMI | 242 | 369 | 482 | 532 | 468 | 567 | 438 | 326 | 222 | 355 | 169 | 11 | 4181 |
| Waist/hip ratio | 240 | 367 | 449 | 449 | 286 | 336 | 301 | 227 | 174 | 321 | 169 | 11 | 3330 |
| Smoker | 230 | 343 | 479 | 527 | 467 | 557 | 305 | 220 | 139 | 354 | 164 | 11 | 3796 |
| FH of HD | 228 | 342 | 480 | 526 | 463 | 467 | 377 | 434 | 389 | 431 | 407 | 37 | 4581 |
| RBC | 242 | 368 | 481 | 506 | 402 | 461 | 365 | 283 | 210 | 332 | 169 | 11 | 3830 |
| WBC | 242 | 368 | 481 | 501 | 392 | 437 | 355 | 276 | 209 | 332 | 169 | 11 | 3773 |
| PLT | 242 | 368 | 480 | 493 | 370 | 416 | 344 | 268 | 205 | 332 | 169 | 11 | 3698 |
| Hemoglobin | 242 | 368 | 481 | 506 | 402 | 461 | 365 | 283 | 210 | 332 | 169 | 11 | 3830 |
| HDL-C | 242 | 368 | 481 | 505 | 404 | 468 | 369 | 286 | 211 | 334 | 169 | 11 | 3848 |
| LDL-C | 242 | 368 | 481 | 493 | 375 | 410 | 345 | 260 | 197 | 328 | 169 | 11 | 3679 |
| Triglycerides | 242 | 368 | 481 | 506 | 406 | 469 | 370 | 286 | 211 | 334 | 169 | 11 | 3853 |
| HbA1c | 242 | 367 | 481 | 487 | 373 | 437 | 354 | 280 | 205 | 333 | 169 | 11 | 3739 |
| HOMA-IR | 242 | 367 | 481 | 451 | 285 | 335 | 303 | 228 | 172 | 320 | 169 | 11 | 3364 |
| AST | 242 | 368 | 481 | 505 | 406 | 472 | 366 | 285 | 210 | 334 | 169 | 11 | 3849 |
| ALT | 242 | 368 | 481 | 508 | 408 | 476 | 366 | 285 | 210 | 334 | 169 | 11 | 3858 |
| γ-GTP | 242 | 368 | 481 | 507 | 408 | 476 | 367 | 285 | 210 | 334 | 169 | 11 | 3858 |
| Comorbidity score | 231 | 346 | 481 | 527 | 467 | 558 | 392 | 404 | 390 | 433 | 409 | 38 | 4676 |
| Good sleep status | 230 | 342 | 478 | 526 | 465 | 557 | 305 | 219 | 138 | 353 | 164 | 11 | 3788 |
| Hand grip strength | 242 | 366 | 481 | 450 | 282 | 336 | 302 | 229 | 171 | 314 | 166 | 11 | 3350 |
| Leg power | 235 | 352 | 461 | 436 | 273 | 346 | 280 | 208 | 165 | 303 | 160 | 11 | 3230 |
| Seated forward bend | 242 | 365 | 480 | 448 | 283 | 336 | 302 | 226 | 172 | 316 | 166 | 11 | 3347 |
| Step counts | 225 | 343 | 454 | 523 | 466 | 564 | 386 | 258 | 220 | 352 | 148 | 1 | 3940 |
| n-3/n-6 FA ratio | 223 | 359 | 481 | 528 | 463 | 566 | 438 | 323 | 221 | 353 | 169 | 11 | 4135 |
| SFA intake | 223 | 359 | 481 | 528 | 463 | 566 | 438 | 323 | 221 | 353 | 169 | 11 | 4135 |
| Alcohol consumption | 223 | 359 | 481 | 528 | 463 | 566 | 438 | 323 | 221 | 353 | 169 | 11 | 4135 |
| Salt intake | 223 | 359 | 481 | 528 | 463 | 566 | 438 | 323 | 221 | 353 | 169 | 11 | 4135 |
| Sugar intake | 223 | 359 | 481 | 528 | 463 | 566 | 438 | 323 | 221 | 353 | 169 | 11 | 4135 |
| Meat intake | 223 | 359 | 481 | 528 | 463 | 566 | 438 | 323 | 221 | 353 | 169 | 11 | 4135 |
| FV intake | 223 | 359 | 481 | 528 | 463 | 566 | 438 | 323 | 221 | 353 | 169 | 11 | 4135 |
| Pulses intake | 223 | 359 | 481 | 528 | 463 | 566 | 438 | 323 | 221 | 353 | 169 | 11 | 4135 |

The variables are shown as number of participants; the number of individuals for whom we could obtain information on the status of any of the 10 types of comorbidities (including hypertension, dyslipidemia, diabetes, ischemic heart disease, other heart diseases, cerebrovascular diseases, renal failure, cancer, osteoporosis, and depression). ABI, ankle-brachial index, ALT, alanine aminotransferase; AST, aspartate aminotransferase; BF, blood flow; BMI, body mass index; BVAI, biological vascular aging indicator; DD, common carotid diastolic diameter, FA, fatty acid; FH, family history; FV, fruits and vegetables; HbA1c, hemoglobin A1C; HD, heart disease; HDL-C, high density lipoprotein cholesterol; HOMA-IR, homeostasis model assessment of insulin resistance; HR, heart rate; LDL-C, low density lipoprotein cholesterol; MBV, carotid artery mean blood velocity, MT, measurement times; PLT, platelets; PWV, carotid-femoral pulse wave velocity, RBC, red blood count; SBP, systolic blood pressure; SFA, saturated fatty acid; VI, vascular aging index, WBC, white blood cell count; γ-GTP, γ-glutamyl transpeptidase

**Supplemental Table 2**. Assessment of multicollinearity by vascular aging index for the multivariate panel data analysis

|  | FAS | | | | | | | | | BCC | CC |
| --- | --- | --- | --- | --- | --- | --- | --- | --- | --- | --- | --- |
|  | SBP | ABI | HR | DD | MBV | BF | IMT | PWV | VI |  |  |
| Age | 3.04 | 3.19 | 3.19 | 3.15 | 3.10 | 3.14 | 3.15 | 3.20 | 3.15 | 3.15 | 3.12 |
| Female sex | 5.21 | 5.21 | 5.21 | 5.19 | 5.30 | 5.19 | 5.19 | 5.19 | 5.17 | 5.17 | 5.25 |
| Local area | 1.86 | 1.91 | 1.91 | 1.85 | 1.95 | 1.84 | 1.85 | 1.92 | 1.85 | 1.85 | 1.86 |
| BMI | 2.19 | 2.16 | 2.16 | 2.19 | 2.21 | 2.21 | 2.19 | 2.15 | 2.19 | 2.19 | 2.15 |
| Waist/hip ratio | 2.31 | 2.24 | 2.24 | 2.26 | 2.25 | 2.26 | 2.26 | 2.23 | 2.25 | 2.25 | 2.23 |
| Smoker | 1.37 | 1.35 | 1.35 | 1.33 | 1.33 | 1.32 | 1.33 | 1.35 | 1.32 | 1.32 | 1.30 |
| FH of HD | 1.04 | 1.05 | 1.05 | 1.05 | 1.04 | 1.05 | 1.05 | 1.04 | 1.05 | 1.05 | 1.05 |
| RBC | 1.90 | 3.17 | 3.17 | 3.11 | 3.16 | 3.11 | 3.11 | 3.17 | 3.12 | 3.12 | 3.15 |
| WBC | 1.24 | 1.32 | 1.32 | 1.32 | 1.29 | 1.32 | 1.32 | 1.30 | 1.31 | 1.31 | 1.30 |
| PLT | 1.07 | 1.18 | 1.18 | 1.25 | 1.17 | 1.25 | 1.25 | 1.18 | 1.25 | 1.25 | 1.24 |
| Hemoglobin | 1.33 | 3.58 | 3.58 | 3.56 | 3.56 | 3.55 | 3.56 | 3.61 | 3.59 | 3.58 | 3.60 |
| HDL-C | 1.56 | 1.58 | 1.58 | 1.59 | 1.58 | 1.58 | 1.58 | 1.58 | 1.58 | 1.58 | 1.60 |
| LDL-C | 1.21 | 1.21 | 1.21 | 1.23 | 1.22 | 1.23 | 1.23 | 1.21 | 1.23 | 1.23 | 1.23 |
| Triglycerides | 1.44 | 1.45 | 1.45 | 1.46 | 1.46 | 1.46 | 1.46 | 1.46 | 1.47 | 1.47 | 1.55 |
| HbA1c | 1.01 | 1.72 | 1.72 | 1.67 | 1.66 | 1.66 | 1.67 | 1.69 | 1.64 | 1.64 | 1.66 |
| HOMA-IR | 1.33 | 1.41 | 1.41 | 1.49 | 1.51 | 1.50 | 1.49 | 1.41 | 1.49 | 1.49 | 1.49 |
| AST | 2.70 | 2.74 | 2.74 | 2.77 | 2.75 | 2.77 | 2.77 | 2.73 | 2.77 | 2.77 | 2.76 |
| ALT | 3.23 | 3.33 | 3.33 | 3.39 | 3.36 | 3.39 | 3.39 | 3.33 | 3.39 | 3.39 | 3.34 |
| γ-GTP | 1.53 | 1.54 | 1.54 | 1.55 | 1.54 | 1.55 | 1.55 | 1.54 | 1.55 | 1.56 | 1.58 |
| Comorbidity score | 1.45 | 1.54 | 1.54 | 1.57 | 1.53 | 1.55 | 1.57 | 1.52 | 1.54 | 1.54 | 1.53 |
| Good sleep status | 1.06 | 1.06 | 1.06 | 1.06 | 1.06 | 1.06 | 1.06 | 1.06 | 1.06 | 1.06 | 1.06 |
| Hand grip strength | 5.21 | 4.96 | 4.96 | 4.90 | 4.99 | 4.89 | 4.90 | 4.99 | 4.91 | 4.90 | 4.82 |
| Leg power | 5.21 | 4.96 | 4.96 | 4.90 | 5.05 | 4.88 | 4.89 | 4.98 | 4.90 | 4.89 | 5.09 |
| Seated forward bend | 1.44 | 1.43 | 1.43 | 1.41 | 1.43 | 1.42 | 1.41 | 1.42 | 1.41 | 1.41 | 1.43 |
| Step counts | 1.20 | 1.21 | 1.21 | 1.21 | 1.21 | 1.20 | 1.21 | 1.20 | 1.19 | 1.19 | 1.18 |
| n-3/n-6 FA ratio | 1.48 | 1.50 | 1.50 | 1.49 | 1.49 | 1.49 | 1.49 | 1.51 | 1.50 | 1.50 | 1.49 |
| SFA intake | 1.46 | 1.47 | 1.47 | 1.48 | 1.47 | 1.49 | 1.48 | 1.46 | 1.48 | 1.49 | 1.49 |
| Alcohol consumption | 1.61 | 1.62 | 1.62 | 1.63 | 1.63 | 1.63 | 1.63 | 1.63 | 1.64 | 1.64 | 1.63 |
| Salt intake | 1.49 | 1.51 | 1.51 | 1.51 | 1.50 | 1.51 | 1.51 | 1.52 | 1.52 | 1.52 | 1.51 |
| Sugar intake | 1.37 | 1.37 | 1.37 | 1.38 | 1.38 | 1.38 | 1.38 | 1.37 | 1.38 | 1.38 | 1.39 |
| Meat intake | 1.33 | 1.32 | 1.32 | 1.32 | 1.31 | 1.32 | 1.32 | 1.31 | 1.31 | 1.32 | 1.33 |
| FV intake | 1.60 | 1.60 | 1.60 | 1.59 | 1.59 | 1.59 | 1.59 | 1.60 | 1.60 | 1.59 | 1.59 |
| Pulses intake | 1.26 | 1.26 | 1.26 | 1.27 | 1.27 | 1.27 | 1.27 | 1.26 | 1.27 | 1.27 | 1.27 |

The values are shown as variance inflation factor (VIF). ABI, ankle-brachial index, ALT, alanine aminotransferase; AST, aspartate aminotransferase; BCC, biological vascular aging indicator complete case; BF, blood flow; BMI, body mass index; CC, complete case; DD, common carotid diastolic diameter, FA, fatty acid; FAS, full analysis set; FH, family history; FV, fruits and vegetables; HbA1c, hemoglobin A1C; HD, heart disease; HDL-C, high density lipoprotein cholesterol; HOMA-IR, homeostasis model assessment of insulin resistance; HR, heart rate; LDL-C, low density lipoprotein cholesterol; MBV, carotid artery mean blood velocity, MT, measurement times; PLT, platelets; PWV, carotid-femoral pulse wave velocity, RBC, red blood count; SBP, systolic blood pressure; SFA, saturated fatty acid; VI, vascular aging index, WBC, white blood cell count; γ-GTP, γ-glutamyl transpeptidase

**Supplemental Table 3**. Number and characteristics of baseline measurements of individuals with information on all covariates

|  | Baseline and follow-up measurements in individuals | | | | | | | | | | | | | |
| --- | --- | --- | --- | --- | --- | --- | --- | --- | --- | --- | --- | --- | --- | --- |
|  | FAS | | | | | BCC | | | |  | CC | | | |
|  | *n* | MT | Distribution | |  | *n* | MT | Distribution | |  | *n* | MT | Distribution | |
| Age [years] | 697 | 4880 | 52.5 | (11.5) |  | 678 | 2936 | 52.6 | (11.5) |  | 648 | 2633 | 52.7 | (11.5) |
| Women [n (%)] | 697 | 4880 | 485 | (69.6) |  | 678 | 2936 | 473 | (69.8) |  | 648 | 2633 | 451 | (69.6) |
| Local area [n (%)] | 697 | 4880 | 232 | (33.3) |  | 678 | 2936 | 219 | (32.3) |  | 648 | 2633 | 199 | (30.7) |
| Body mass index [kg/m^2^] | 697 | 4181 | 22.5 | (2.9) |  | 678 | 2936 | 22.5 | (2.9) |  | 648 | 2633 | 22.5 | (2.9) |
| Waist/hip ratio | 697 | 3330 | 0.88 | (0.07) |  | 678 | 2914 | 0.88 | (0.07) |  | 648 | 2633 | 0.88 | (0.07) |
| Smoker [n (%)] | 697 | 3796 | 198 | (28.4) |  | 678 | 2781 | 188 | (27.7) |  | 648 | 2633 | 182 | (28.1) |
| FH of HD [n (%)] | 697 | 4581 | 455 | (65.3) |  | 678 | 2908 | 445 | (65.6) |  | 648 | 2633 | 425 | (65.6) |
| RBC [count 10^12^/L] | 697 | 3830 | 451 | (39) |  | 678 | 2931 | 450 | (39) |  | 648 | 2633 | 450 | (38) |
| WBC [count 10^9^/L] | 697 | 3773 | 4995 | (1257) |  | 678 | 2931 | 4995 | (1250) |  | 648 | 2633 | 4982 | (1245) |
| PLT [count 10^9^/L] | 697 | 3698 | 24.1 | (5.3) |  | 678 | 2926 | 24.1 | (5.3) |  | 648 | 2633 | 23.9 | (5.3) |
| Hemoglobin [mg/dL] | 697 | 3830 | 13.6 | (1.4) |  | 678 | 2931 | 13.6 | (1.4) |  | 648 | 2633 | 13.6 | (1.4) |
| HDL-C [mg/dL] | 697 | 3848 | 66 | (17) |  | 678 | 2932 | 66 | (17) |  | 648 | 2633 | 66 | (17) |
| LDL-C [mg/dL] | 697 | 3679 | 125 | (30) |  | 678 | 2931 | 126 | (30) |  | 648 | 2633 | 126 | (30) |
| Triglycerides [mg/dL] | 697 | 3853 | 92 | (57) |  | 678 | 2932 | 92 | (57) |  | 648 | 2633 | 92 | (57) |
| HbA1c [%] | 697 | 3739 | 5.4 | (0.4) |  | 678 | 2931 | 5.4 | (0.5) |  | 648 | 2633 | 5.4 | (0.5) |
| HOMA-IR | 697 | 3364 | 1.03 | (0.74) |  | 678 | 2930 | 1.04 | (0.75) |  | 648 | 2633 | 1.04 | (0.76) |
| AST [IU/L] | 697 | 3849 | 22 | (10) |  | 678 | 2931 | 22 | (10) |  | 648 | 2633 | 22 | (10) |
| ALT [U/L] | 697 | 3858 | 20 | (17) |  | 678 | 2932 | 20 | (16) |  | 648 | 2633 | 20 | (16) |
| γ-GTP [IU/L] | 697 | 3858 | 30 | (38) |  | 678 | 2932 | 30 | (37) |  | 648 | 2633 | 30 | (38) |
| Comorbidity score, 0 score [n (%)] | 697 | 4676 | 529 | (75.9) |  | 678 | 2914 | 516 | (76.1) |  | 648 | 2633 | 492 | (75.9) |
| Good sleep status [n (%)] | 697 | 3788 | 189 | (27.1) |  | 678 | 2778 | 185 | (27.3) |  | 648 | 2633 | 179 | (27.6) |
| Hand grip strength [kg] | 697 | 3350 | 31.6 | (9.0) |  | 678 | 2921 | 31.2 | (8.9) |  | 648 | 2633 | 31.2 | (9.0) |
| Leg power [w] | 697 | 3230 | 1021 | (429) |  | 678 | 2712 | 1002 | (430) |  | 648 | 2633 | 1006 | (432) |
| Seated forward bend [cm] | 697 | 3347 | 38.9 | (9.8) |  | 678 | 2917 | 38.9 | (9.7) |  | 648 | 2633 | 38.9 | (9.8) |
| Step counts [steps/day] | 697 | 3940 | 10205 | (3542) |  | 678 | 2851 | 10209 | (3495) |  | 648 | 2633 | 10262 | (3491) |
| n-3/n-6 FA ratio | 697 | 4135 | 0.26 | (0.07) |  | 678 | 2900 | 0.26 | (0.07) |  | 648 | 2633 | 0.26 | (0.07) |
| SFA intake [% energy/day] | 697 | 4135 | 7.0 | (1.8) |  | 678 | 2900 | 7.0 | (1.8) |  | 648 | 2633 | 7.0 | (1.8) |
| Alcohol consumption [% energy/day] | 697 | 4135 | 4.4 | (7.1) |  | 678 | 2900 | 4.3 | (7.1) |  | 648 | 2633 | 4.3 | (7.2) |
| Salt intake [g/1,000 kcal/day] | 697 | 4135 | 5.6 | (1.1) |  | 678 | 2900 | 5.6 | (1.1) |  | 648 | 2633 | 5.6 | (1.1) |
| Sugar intake [% energy/day] | 697 | 4135 | 3.0 | (1.8) |  | 678 | 2900 | 3.0 | (1.8) |  | 648 | 2633 | 3.0 | (1.8) |
| Meat intake [g/1,000 kcal/day] | 697 | 4135 | 36.6 | (17.5) |  | 678 | 2900 | 36.4 | (17.1) |  | 648 | 2633 | 36.6 | (17.0) |
| FV intake [g/1,000 kcal/day] | 697 | 4135 | 222 | (105) |  | 678 | 2900 | 228 | (104) |  | 648 | 2633 | 226 | (104) |
| Pulses intake [g/1,000 kcal/day] | 697 | 4135 | 35.6 | (22.2) |  | 678 | 2900 | 36.2 | (22.4) |  | 648 | 2633 | 36.0 | (22.1) |

The variables are presented as mean (standard deviation) or number of cases (percentage). FAS refers to using all the acquired data. BCC refers to the use of the data on all nine BVAIs acquired at the same in-person testing (no missing data exclusively for BVAIs). CC refers to the use of data obtained at the same in-person testing for all covariates included in the multivariate analysis (no missing data for any covariates).

ALT, alanine aminotransferase; AST, aspartate aminotransferase; BCC, biological vascular aging indicator complete case; ; CC, complete case; FAS, full analysis set; FH, family history; FV, fruits and vegetables; HbA1c, hemoglobin A1C; HD, heart disease; HDL-C, high-density lipoprotein cholesterol; HOMA-IR, homeostasis model assessment of insulin resistance; LDL-C, low-density lipoprotein cholesterol; MT, measurement times; PLT, platelets; RBC, red blood cell count; SFA, saturated fatty acid; WBC, white blood cell count; γ-GTP, γ-glutamyl transpeptidase

**Supplemental Table 4**. Accuracy and precision of BVAIs in all participants

|  | BVAIs (*n* = 678) [2,936 measurements] | | | | | | | | |
| --- | --- | --- | --- | --- | --- | --- | --- | --- | --- |
|  | SBP [mmHg] | ABI | HR [bpm] | DD [mm] | MBV [cm/s] | BF [mL/min] | IMT [mm] | PWV [cm/s] | VI |
| Mean (SD) | 120 (14) | 1.15 (0.08) | 63 (9) | 6.2 (0.7) | 28.8 (6.3) | 536 (124) | 0.68 (0.12) | 934 (212) | 35.5 (5.8) |
| CV_w_ [%] | 5.4 | 3.8 | 8.7 | 3.0 | 13.9 | 14.6 | 8.2 | 11.0 | 7.4 |
| CV_b_ [%] | 9.4 | 4.1 | 11.9 | 8.9 | 17.5 | 18.6 | 14.5 | 16.7 | 12.3 |
| VR | 0.57 | 0.92 | 0.74 | 0.34 | 0.79 | 0.79 | 0.56 | 0.66 | 0.60 |
| ICC | 0.64 | 0.52 | 0.58 | 0.75 | 0.56 | 0.56 | 0.64 | 0.60 | 0.63 |
| Required group size |  |  |  |  |  |  |  |  |  |
| Specified % deviation |  |  |  |  |  |  |  |  |  |
| 2.5 | 73 | 19 | 134 | 54 | 307 | 343 | 171 | 247 | 127 |
| 5 | 18 | 5 | 33 | 13 | 77 | 86 | 43 | 62 | 32 |
| 10 | 5 | 1 | 8 | 3 | 19 | 21 | 11 | 15 | 8 |
| Required survey times |  |  |  |  |  |  |  |  |  |
| Specified *r* |  |  |  |  |  |  |  |  |  |
| 0.85 | 1 | 2 | 2 | 1 | 2 | 2 | 1 | 2 | 2 |
| 0.90 | 2 | 4 | 3 | 1 | 3 | 3 | 2 | 3 | 3 |
| 0.95 | 5 | 9 | 7 | 3 | 7 | 7 | 5 | 6 | 6 |
| Required survey times |  |  |  |  |  |  |  |  |  |
| Specified % deviation |  |  |  |  |  |  |  |  |  |
| 5 | 4 | 2 | 12 | 1 | 30 | 33 | 10 | 19 | 8 |
| 10 | 1 | 1 | 3 | 0 | 7 | 8 | 3 | 5 | 2 |
| 20 | 0 | 0 | 1 | 0 | 2 | 2 | 1 | 1 | 1 |

ABI, ankle brachial index; BF, blood flow; BVAI, biological vascular aging indicator; CV_w_, coefficient of within-person variance; CV_b_, coefficient of between-person variance; DD, common-carotid diastolic diameter; HR, heart rate; ICC, intraclass correlation coefficient; IMT, common carotid intima-media thickness; MBV, mean blood velocity; PWV, carotid-femoral pulse wave velocity; *r*, correlation coefficient; SBP, systolic blood pressure; SD, standard deviation; VI, vascular aging index; VR, within-person/between-person variance ratio

**Supplemental Table 5**. Accuracy and precision of BVAIs in men

|  | BVAIs (*n* = 205) [732 measurements] | | | | | | | | |
| --- | --- | --- | --- | --- | --- | --- | --- | --- | --- |
|  | SBP [mmHg] | ABI | HR [bpm] | DD [mm] | MBV [cm/s] | BF [mL/min] | IMT [mm] | PWV [cm/s] | VI |
| Mean (SD) | 119 (13) | 1.16 (0.08) | 63 (10) | 6.1 (0.7) | 28.2 (6.5) | 511 (120) | 0.65 (0.12) | 908 (179) | 34.5 (5.0) |
| CV_w_ [%] | 4.9 | 3.9 | 9.6 | 2.9 | 15.3 | 16.3 | 7.7 | 10.9 | 7.1 |
| CV_b_ [%] | 8.7 | 3.7 | 12.8 | 8.3 | 18.2 | 19.2 | 15.1 | 15.6 | 11.9 |
| VR | 0.56 | 1.07 | 0.76 | 0.34 | 0.84 | 0.85 | 0.51 | 0.70 | 0.60 |
| ICC | 0.64 | 0.48 | 0.57 | 0.74 | 0.54 | 0.54 | 0.66 | 0.59 | 0.62 |
| Required group size |  |  |  |  |  |  |  |  |  |
| Specified % deviation |  |  |  |  |  |  |  |  |  |
| 2.5 | 61 | 18 | 157 | 48 | 348 | 389 | 177 | 223 | 118 |
| 5 | 15 | 4 | 39 | 12 | 87 | 97 | 44 | 56 | 29 |
| 10 | 4 | 1 | 10 | 3 | 22 | 24 | 11 | 14 | 7 |
| Required survey times |  |  |  |  |  |  |  |  |  |
| Specified *r* |  |  |  |  |  |  |  |  |  |
| 0.85 | 1 | 3 | 2 | 1 | 2 | 2 | 1 | 2 | 2 |
| 0.90 | 2 | 5 | 3 | 1 | 4 | 4 | 2 | 3 | 3 |
| 0.95 | 5 | 10 | 7 | 3 | 8 | 8 | 5 | 6 | 6 |
| Required survey times |  |  |  |  |  |  |  |  |  |
| Specified % deviation |  |  |  |  |  |  |  |  |  |
| 5 | 4 | 2 | 14 | 1 | 36 | 41 | 9 | 18 | 8 |
| 10 | 1 | 1 | 4 | 0 | 9 | 10 | 2 | 5 | 2 |
| 20 | 0 | 0 | 1 | 0 | 2 | 3 | 1 | 1 | 0 |

ABI, ankle brachial index; BF, blood flow; BVAI, biological vascular aging indicator; CV_w_, coefficient of within-person variance; CV_b_, coefficient of between-person variance; DD, common-carotid diastolic diameter; HR, heart rate; ICC, intraclass correlation coefficient; IMT, common carotid intima-media thickness; MBV, mean blood velocity; PWV, carotid-femoral pulse wave velocity; *r*, correlation coefficient; SBP, systolic blood pressure; SD, standard deviation; VI, vascular aging index; VR, within-person/between-person variance ratio

**Supplemental Table 6**. Accuracy and precision of BVAIs in women

|  | BVAIs (*n* = 473) [2,204 measurements] | | | | | | | | |
| --- | --- | --- | --- | --- | --- | --- | --- | --- | --- |
|  | SBP [mmHg] | ABI | HR [bpm] | DD [mm] | MBV [cm/s] | BF [mL/min] | IMT [mm] | PWV [cm/s] | VI |
| Mean (SD) | 121 (15) | 1.15 (0.07) | 63 (9) | 6.2 (0.7) | 29.1 (6.2) | 548 (125) | 0.69 (0.12) | 947 (226) | 36.0 (6.1) |
| CV_w_ [%] | 5.6 | 3.7 | 8.3 | 3.1 | 13.2 | 13.8 | 8.4 | 11.1 | 7.5 |
| CV_b_ [%] | 9.8 | 4.3 | 11.5 | 9.1 | 17.2 | 18.3 | 14.2 | 17.3 | 12.5 |
| VR | 0.58 | 0.86 | 0.73 | 0.34 | 0.77 | 0.76 | 0.59 | 0.64 | 0.60 |
| ICC | 0.63 | 0.54 | 0.58 | 0.75 | 0.56 | 0.57 | 0.63 | 0.61 | 0.63 |
| Required group size |  |  |  |  |  |  |  |  |  |
| Specified % deviation |  |  |  |  |  |  |  |  |  |
| 2.5 | 79 | 20 | 123 | 57 | 289 | 322 | 168 | 259 | 131 |
| 5 | 20 | 5 | 31 | 14 | 72 | 81 | 42 | 65 | 33 |
| 10 | 5 | 1 | 8 | 4 | 18 | 20 | 11 | 16 | 8 |
| Required survey times |  |  |  |  |  |  |  |  |  |
| Specified *r* |  |  |  |  |  |  |  |  |  |
| 0.85 | 1 | 2 | 2 | 1 | 2 | 2 | 2 | 2 | 2 |
| 0.90 | 2 | 4 | 3 | 1 | 3 | 3 | 3 | 3 | 3 |
| 0.95 | 5 | 8 | 7 | 3 | 7 | 7 | 5 | 6 | 6 |
| Required survey times |  |  |  |  |  |  |  |  |  |
| Specified % deviation |  |  |  |  |  |  |  |  |  |
| 5 | 5 | 2 | 11 | 1 | 27 | 29 | 11 | 19 | 9 |
| 10 | 1 | 1 | 3 | 0 | 7 | 7 | 3 | 5 | 2 |
| 20 | 0 | 0 | 1 | 0 | 2 | 2 | 1 | 1 | 1 |

ABI, ankle brachial index; BF, blood flow; BVAI, biological vascular aging indicator; CV_w_, coefficient of within-person variance; CV_b_, coefficient of between-person variance; DD, common-carotid diastolic diameter; HR, heart rate; ICC, intraclass correlation coefficient; IMT, common carotid intima-media thickness; MBV, mean blood velocity; PWV, carotid-femoral pulse wave velocity; *r*, correlation coefficient; SBP, systolic blood pressure; SD, standard deviation; VI, vascular aging index; VR, within-person/between-person variance ratio

**Supplemental Table 7**. Multivariate panel data analysis for SBP and vascular aging–related covariate trajectory

|  | FAS (*n* = 690) [Measurements = 3,636] | | | | | | | BCC (*n* = 678) [Measurements = 2,936] | | | | | | | CC (*n* = 648) [Measurements = 2,633] | | | | | | |
| --- | --- | --- | --- | --- | --- | --- | --- | --- | --- | --- | --- | --- | --- | --- | --- | --- | --- | --- | --- | --- | --- |
|  | RC | 95% CI | | | | | z score | RC | 95% CI | | | | | z score | RC | 95% CI | | | | | z score |
| Variables | *R^2^* = 0.235 | | | | | | | *R^2^* = 0.256 | | | | | | | *R^2^* = 0.250 | | | | | | |
| Age [1-year increment] | 0.503 | ( | 0.419 | – | 0.586 | ) | **11.77** | 0.511 | ( | 0.420 | – | 0.602 | ) | **10.99** | 0.465 | ( | 0.381 | – | 0.549 | ) | **10.38** |
| Female sex | -2.450 | ( | -5.525 | – | 0.626 | ) | -1.56 | -1.205 | ( | -4.538 | – | 2.128 | ) | -0.71 | 0.390 | ( | -2.566 | – | 3.346 | ) | 0.19 |
| Local area | 0.553 | ( | -1.574 | – | 2.680 | ) | 0.51 | 1.154 | ( | -1.101 | – | 3.410 | ) | 1.00 | 1.246 | ( | -0.905 | – | 3.397 | ) | 1.46 |
| BMI [1 kg/m^2^ increment] | 1.288 | ( | 0.977 | – | 1.599 | ) | **8.13** | 1.259 | ( | 0.927 | – | 1.590 | ) | **7.43** | 1.170 | ( | 0.842 | – | 1.498 | ) | **7.03** |
| Waist/hip ratio [0.1-point increment] | -0.670 | ( | -1.663 | – | 0.323 | ) | -1.32 | -0.825 | ( | -1.876 | – | 0.226 | ) | -1.54 | -0.373 | ( | -1.422 | – | 0.677 | ) | -0.85 |
| Smoker | -0.029 | ( | -1.559 | – | 1.501 | ) | -0.04 | 0.245 | ( | -1.419 | – | 1.909 | ) | 0.29 | 0.408 | ( | -1.377 | – | 2.194 | ) | 0.42 |
| FH of HD | -0.601 | ( | -1.467 | – | 0.265 | ) | -1.36 | -0.518 | ( | -1.507 | – | 0.471 | ) | -1.03 | -0.293 | ( | -1.309 | – | 0.723 | ) | -0.42 |
| RBC [10 count 10^12^/L increment] | 0.362 | ( | 0.182 | – | 0.542 | ) | **3.95** | 0.309 | ( | 0.047 | – | 0.571 | ) | **2.31** | 0.363 | ( | 0.105 | – | 0.622 | ) | **2.72** |
| WBC [10 count 10^9^/L increment] | -0.001 | ( | -0.005 | – | 0.003 | ) | -0.66 | 0.000 | ( | -0.004 | – | 0.004 | ) | -0.04 | -0.001 | ( | -0.005 | – | 0.003 | ) | -0.40 |
| PLT [1 count 10^9^/L increment] | -0.022 | ( | -0.066 | – | 0.021 | ) | -1.02 | -0.065 | ( | -0.185 | – | 0.055 | ) | -1.07 | -0.057 | ( | -0.176 | – | 0.063 | ) | -1.22 |
| Hemoglobin [1 mg/dL increment] | -0.006 | ( | -0.175 | – | 0.163 | ) | -0.07 | 0.098 | ( | -0.639 | – | 0.835 | ) | 0.26 | 0.119 | ( | -0.611 | – | 0.849 | ) | 0.13 |
| HDL-C [1 mg/dL increment] | 0.060 | ( | 0.021 | – | 0.099 | ) | **3.02** | 0.045 | ( | 0.003 | – | 0.088 | ) | **2.09** | 0.064 | ( | 0.022 | – | 0.106 | ) | **2.80** |
| LDL-C [1 mg/dL increment] | 0.018 | ( | 0.001 | – | 0.036 | ) | **2.12** | 0.014 | ( | -0.005 | – | 0.032 | ) | 1.48 | -0.001 | ( | -0.020 | – | 0.018 | ) | 0.08 |
| Triglycerides [1 mg/dL increment] | 0.017 | ( | 0.010 | – | 0.025 | ) | **4.59** | 0.016 | ( | 0.008 | – | 0.024 | ) | **3.96** | 0.019 | ( | 0.009 | – | 0.029 | ) | **4.00** |
| HbA1c [1 % increment] | 0.005 | ( | -0.033 | – | 0.042 | ) | 0.23 | 2.073 | ( | 0.720 | – | 3.426 | ) | **3.00** | 1.553 | ( | 0.134 | – | 2.971 | ) | **2.14** |
| HOMA-IR [1-point increment] | -0.104 | ( | -0.636 | – | 0.429 | ) | -0.38 | -0.705 | ( | -1.376 | – | -0.035 | ) | **-2.06** | -0.855 | ( | -1.577 | – | -0.133 | ) | **-2.35** |
| AST [1 IU/L increment] | 0.070 | ( | -0.026 | – | 0.165 | ) | 1.43 | 0.056 | ( | -0.042 | – | 0.153 | ) | 1.11 | 0.063 | ( | -0.033 | – | 0.159 | ) | 1.14 |
| ALT [1 IU/L increment] | -0.018 | ( | -0.085 | – | 0.049 | ) | -0.54 | 0.001 | ( | -0.069 | – | 0.070 | ) | 0.01 | -0.015 | ( | -0.084 | – | 0.053 | ) | -0.36 |
| γ-GTP [1 IU/L increment] | 0.001 | ( | -0.020 | – | 0.023 | ) | 0.10 | -0.005 | ( | -0.028 | – | 0.018 | ) | -0.45 | 0.009 | ( | -0.015 | – | 0.033 | ) | 0.65 |
| Comorbidity score [1-point increment] | -0.457 | ( | -1.075 | – | 0.160 | ) | -1.45 | -0.685 | ( | -1.361 | – | -0.008 | ) | **-1.98** | -0.390 | ( | -1.105 | – | 0.325 | ) | -1.28 |
| Good sleep status | 0.191 | ( | -0.770 | – | 1.151 | ) | 0.39 | 0.061 | ( | -0.957 | – | 1.080 | ) | 0.12 | -0.080 | ( | -1.118 | – | 0.957 | ) | 0.01 |
| Hand grip strength [1 kg increment] | 0.196 | ( | 0.064 | – | 0.328 | ) | **2.91** | 0.217 | ( | 0.076 | – | 0.359 | ) | **3.01** | 0.272 | ( | 0.136 | – | 0.407 | ) | **3.75** |
| Leg power [10 w increment] | -0.015 | ( | -0.039 | – | 0.009 | ) | -1.25 | -0.007 | ( | -0.032 | – | 0.017 | ) | -0.59 | 0.003 | ( | -0.022 | – | 0.028 | ) | -0.01 |
| Seated forward bend [1 cm increment] | -0.031 | ( | -0.092 | – | 0.029 | ) | -1.02 | -0.039 | ( | -0.106 | – | 0.027 | ) | -1.16 | -0.061 | ( | -0.128 | – | 0.006 | ) | -1.49 |
| Step counts [1000 steps/day increment] | 0.027 | ( | -0.125 | – | 0.179 | ) | 0.35 | 0.067 | ( | -0.091 | – | 0.226 | ) | 0.83 | 0.109 | ( | -0.049 | – | 0.267 | ) | 1.49 |
| n-3/n-6 FA ratio [1-point increment] | 0.199 | ( | -6.715 | – | 7.113 | ) | 0.06 | 0.899 | ( | -6.542 | – | 8.340 | ) | 0.24 | 0.606 | ( | -6.956 | – | 8.168 | ) | -0.09 |
| SFA intake [1 % energy/day increment] | -0.030 | ( | -0.311 | – | 0.250 | ) | -0.21 | -0.036 | ( | -0.328 | – | 0.257 | ) | -0.24 | 0.160 | ( | -0.153 | – | 0.473 | ) | 0.34 |
| Alcohol consumption [1 % energy/day increment] | 0.116 | ( | 0.015 | – | 0.217 | ) | **2.25** | 0.144 | ( | 0.039 | – | 0.249 | ) | **2.69** | 0.138 | ( | 0.032 | – | 0.243 | ) | **2.62** |
| Salt intake [1 g/1,000 kcal/day increment] | 0.269 | ( | -0.146 | – | 0.684 | ) | 1.27 | 0.462 | ( | 0.025 | – | 0.898 | ) | **2.07** | 0.728 | ( | 0.269 | – | 1.187 | ) | **2.63** |
| Sugar intake [1 % energy/day increment] | 0.100 | ( | -0.190 | – | 0.390 | ) | 0.67 | 0.056 | ( | -0.251 | – | 0.363 | ) | 0.36 | 0.000 | ( | -0.310 | – | 0.309 | ) | 0.08 |
| Meat intake [10 g/1,000 kcal/day increment] | 0.186 | ( | -0.072 | – | 0.443 | ) | 1.41 | 0.093 | ( | -0.181 | – | 0.367 | ) | 0.66 | 0.034 | ( | -0.245 | – | 0.314 | ) | -0.06 |
| FV intake [10 g/1,000 kcal/day increment] | -0.043 | ( | -0.091 | – | 0.006 | ) | -1.73 | -0.044 | ( | -0.095 | – | 0.006 | ) | -1.72 | 0.007 | ( | -0.061 | – | 0.076 | ) | -1.12 |
| Pulses intake [10 g/1,000 kcal/day increment] | -0.073 | ( | -0.266 | – | 0.120 | ) | -0.74 | -0.175 | ( | -0.377 | – | 0.027 | ) | -1.70 | -0.177 | ( | -0.383 | – | 0.029 | ) | -1.73 |

The values are shown as regression coefficients and 95% confidence intervals for the effect of SBP per unit increment of each covariate. The z scores indicated in bold are statistically significant (≤-1.96 or ≥1.96). Sex, area, and FH of HD were time-stable variables, while the other covariates were time-varying variables. ALT, alanine aminotransferase; AST, aspartate aminotransferase; BCC, biological vascular aging indicator complete case; BMI, body mass index; CC, complete case; FA, fatty acid; FAS, full analysis set; FH, family history; FV, fruits and vegetables; HbA1c, hemoglobin A1C; HD, heart disease; HDL-C, high density lipoprotein cholesterol; HOMA-IR, homeostasis model assessment of insulin resistance; LDL-C, low density lipoprotein cholesterol; PLT, platelets; RBC, red blood cell count; RC, regression coefficient; SBP, systolic blood pressure; SFA, saturated fatty acid; WBC, white blood cell count; γ-GTP, γ-glutamyl transpeptidase

**Supplemental Table 8**. Multivariate panel data analysis for ABI and vascular aging–related covariate trajectory

|  | FAS (*n* = 686) [Measurements = 3,341] | | | | | | | BCC (*n* = 678) [Measurements = 2,936] | | | | | | | CC (*n* = 648) [Measurements = 2,633] | | | | | | |
| --- | --- | --- | --- | --- | --- | --- | --- | --- | --- | --- | --- | --- | --- | --- | --- | --- | --- | --- | --- | --- | --- |
|  | RC ^-10^2 | 95% CI | | | | | z score | RC | 95% CI | | | | | z score | RC | 95% CI | | | | | z score |
| Variables | *R^2^* = 0.099 | | | | | | | *R^2^* = 0.101 | | | | | | | *R^2^* = 0.099 | | | | | | |
| Age [1-year increment] | 0.076 | ( | 0.031 | – | 0.121 | ) | **3.28** | 0.084 | ( | 0.037 | – | 0.131 | ) | **3.50** | 0.092 | ( | 0.043 | – | 0.141 | ) | **3.66** |
| Female sex | -3.691 | ( | -5.340 | – | -2.041 | ) | **-4.39** | -3.462 | ( | -5.173 | – | -1.751 | ) | **-3.97** | -3.423 | ( | -5.191 | – | -1.655 | ) | **-3.80** |
| Local area | -1.413 | ( | -2.477 | – | -0.349 | ) | **-2.60** | -1.137 | ( | -2.245 | – | -0.029 | ) | **-2.01** | -1.240 | ( | -2.382 | – | -0.097 | ) | **-2.13** |
| BMI [1 kg/m^2^ increment] | 0.181 | ( | 0.019 | – | 0.342 | ) | **2.19** | 0.225 | ( | 0.055 | – | 0.394 | ) | **2.60** | 0.220 | ( | 0.045 | – | 0.394 | ) | **2.47** |
| Waist/hip ratio [0.1-point increment] | 5.839 | ( | 0.283 | – | 11.394 | ) | **2.06** | 7.239 | ( | 1.391 | – | 13.088 | ) | **2.43** | 7.802 | ( | 1.809 | – | 13.794 | ) | **2.55** |
| Smoker | -0.497 | ( | -1.315 | – | 0.322 | ) | -1.19 | -0.303 | ( | -1.173 | – | 0.567 | ) | -0.68 | -0.424 | ( | -1.385 | – | 0.538 | ) | -0.86 |
| FH of HD | 0.288 | ( | -0.203 | – | 0.779 | ) | 1.15 | 0.009 | ( | -0.545 | – | 0.563 | ) | 0.03 | 0.055 | ( | -0.528 | – | 0.638 | ) | 0.19 |
| RBC [10 count 10^12^/L increment] | -0.018 | ( | -0.151 | – | 0.116 | ) | -0.26 | 0.018 | ( | -0.122 | – | 0.158 | ) | 0.25 | -0.008 | ( | -0.152 | – | 0.136 | ) | -0.10 |
| WBC [10 count 10^9^/L increment] | 0.002 | ( | 0.000 | – | 0.004 | ) | 1.64 | 0.001 | ( | -0.001 | – | 0.004 | ) | 0.96 | 0.001 | ( | -0.002 | – | 0.003 | ) | 0.53 |
| PLT [1 count 10^9^/L increment] | -0.006 | ( | -0.047 | – | 0.034 | ) | -0.31 | -0.010 | ( | -0.074 | – | 0.055 | ) | -0.29 | 0.005 | ( | -0.062 | – | 0.071 | ) | 0.13 |
| Hemoglobin [1 mg/dL increment] | -0.647 | ( | -1.022 | – | -0.272 | ) | **-3.38** | -0.698 | ( | -1.101 | – | -0.296 | ) | **-3.40** | -0.583 | ( | -0.997 | – | -0.169 | ) | **-2.76** |
| HDL-C [1 mg/dL increment] | 0.022 | ( | 0.000 | – | 0.044 | ) | **2.00** | 0.026 | ( | 0.003 | – | 0.048 | ) | **2.23** | 0.021 | ( | -0.003 | – | 0.044 | ) | 1.73 |
| LDL-C [1 mg/dL increment] | 0.008 | ( | -0.001 | – | 0.018 | ) | 1.69 | 0.006 | ( | -0.004 | – | 0.017 | ) | 1.23 | 0.005 | ( | -0.006 | – | 0.016 | ) | 0.94 |
| Triglycerides [1 mg/dL increment] | -0.003 | ( | -0.007 | – | 0.001 | ) | -1.37 | -0.003 | ( | -0.007 | – | 0.002 | ) | -1.15 | -0.003 | ( | -0.009 | – | 0.002 | ) | -1.14 |
| HbA1c [1 % increment] | 0.138 | ( | -0.561 | – | 0.837 | ) | 0.39 | 0.012 | ( | -0.731 | – | 0.755 | ) | 0.03 | -0.089 | ( | -0.887 | – | 0.708 | ) | -0.22 |
| HOMA-IR [1-point increment] | -0.247 | ( | -0.545 | – | 0.050 | ) | -1.63 | -0.126 | ( | -0.506 | – | 0.254 | ) | -0.65 | -0.200 | ( | -0.615 | – | 0.216 | ) | -0.94 |
| AST [1 IU/L increment] | 0.007 | ( | -0.046 | – | 0.059 | ) | 0.25 | 0.003 | ( | -0.052 | – | 0.058 | ) | 0.11 | 0.005 | ( | -0.051 | – | 0.060 | ) | 0.16 |
| ALT [1 IU/L increment] | -0.019 | ( | -0.056 | – | 0.018 | ) | -1.00 | -0.017 | ( | -0.056 | – | 0.022 | ) | -0.86 | -0.017 | ( | -0.056 | – | 0.023 | ) | -0.83 |
| γ-GTP [1 IU/L increment] | 0.006 | ( | -0.005 | – | 0.018 | ) | 1.07 | 0.002 | ( | -0.010 | – | 0.014 | ) | 0.30 | 0.002 | ( | -0.011 | – | 0.015 | ) | 0.26 |
| Comorbidity score [1-point increment] | -0.198 | ( | -0.544 | – | 0.148 | ) | -1.12 | -0.205 | ( | -0.584 | – | 0.175 | ) | -1.06 | -0.137 | ( | -0.548 | – | 0.275 | ) | -0.65 |
| Good sleep status | -0.475 | ( | -1.018 | – | 0.069 | ) | -1.71 | -0.473 | ( | -1.050 | – | 0.104 | ) | -1.61 | -0.689 | ( | -1.291 | – | -0.088 | ) | **-2.25** |
| Hand grip strength [1 kg increment] | 0.022 | ( | -0.052 | – | 0.095 | ) | 0.58 | 0.040 | ( | -0.036 | – | 0.117 | ) | 1.03 | 0.038 | ( | -0.040 | – | 0.117 | ) | 0.95 |
| Leg power [10 w increment] | 0.002 | ( | -0.012 | – | 0.016 | ) | 0.29 | -0.001 | ( | -0.015 | – | 0.013 | ) | -0.09 | -0.003 | ( | -0.017 | – | 0.012 | ) | -0.35 |
| Seated forward bend [1 cm increment] | 0.022 | ( | -0.012 | – | 0.056 | ) | 1.26 | 0.025 | ( | -0.011 | – | 0.062 | ) | 1.37 | 0.027 | ( | -0.011 | – | 0.064 | ) | 1.38 |
| Step counts [1000 steps/day increment] | 0.193 | ( | 0.110 | – | 0.276 | ) | **4.55** | 0.194 | ( | 0.107 | – | 0.282 | ) | **4.38** | 0.190 | ( | 0.101 | – | 0.279 | ) | **4.17** |
| n-3/n-6 FA ratio [1-point increment] | 0.311 | ( | -3.568 | – | 4.190 | ) | 0.16 | 0.664 | ( | -3.594 | – | 4.922 | ) | 0.31 | 1.231 | ( | -3.156 | – | 5.618 | ) | 0.55 |
| SFA intake [1 % energy/day increment] | -0.199 | ( | -0.356 | – | -0.041 | ) | **-2.47** | -0.216 | ( | -0.383 | – | -0.050 | ) | **-2.55** | -0.257 | ( | -0.430 | – | -0.084 | ) | **-2.91** |
| Alcohol consumption [1 % energy/day increment] | -0.044 | ( | -0.099 | – | 0.011 | ) | -1.55 | -0.031 | ( | -0.089 | – | 0.027 | ) | -1.06 | -0.039 | ( | -0.099 | – | 0.020 | ) | -1.29 |
| Salt intake [1 g/1,000 kcal/day increment] | 0.098 | ( | -0.137 | – | 0.333 | ) | 0.82 | 0.063 | ( | -0.186 | – | 0.312 | ) | 0.50 | -0.005 | ( | -0.264 | – | 0.253 | ) | -0.04 |
| Sugar intake [1 % energy/day increment] | -0.042 | ( | -0.205 | – | 0.121 | ) | -0.51 | -0.033 | ( | -0.206 | – | 0.140 | ) | -0.38 | -0.024 | ( | -0.202 | – | 0.154 | ) | -0.26 |
| Meat intake [10 g/1,000 kcal/day increment] | 0.010 | ( | -0.138 | – | 0.159 | ) | 0.14 | 0.058 | ( | -0.099 | – | 0.213 | ) | 0.72 | 0.096 | ( | -0.065 | – | 0.257 | ) | 1.17 |
| FV intake [10 g/1,000 kcal/day increment] | -0.023 | ( | -0.050 | – | 0.005 | ) | -1.62 | -0.026 | ( | -0.055 | – | 0.002 | ) | -1.81 | -0.028 | ( | -0.058 | – | 0.002 | ) | -1.82 |
| Pulses intake [10 g/1,000 kcal/day increment] | 0.113 | ( | 0.004 | – | 0.223 | ) | **2.03** | 0.147 | ( | 0.032 | – | 0.262 | ) | **2.51** | 0.165 | ( | 0.045 | – | 0.285 | ) | **2.70** |

The values are shown as regression coefficients and 95% confidence intervals for the effect of ABI per unit increment of each covariate. The regression coefficients and 95% confidence intervals are shown in the estimated value corrected by 10^2^ because the estimated value was small. The z scores indicated in bold are statistically significant (≤-1.96 or ≥1.96). Sex, area, and FH of HD were time-stable variables, while the other covariates were time-varying variables. Ankle-brachial index; ALT, alanine aminotransferase; AST, aspartate aminotransferase; BCC, biological vascular aging indicator complete case; BMI, body mass index; CC, complete case; FA, fatty acid; FAS, full analysis set; FH, family history; FV, fruits and vegetables; HbA1c, hemoglobin A1C; HD, heart disease; HDL-C, high density lipoprotein cholesterol; HOMA-IR, homeostasis model assessment of insulin resistance; LDL-C, low density lipoprotein cholesterol; PLT, platelets; RBC, red blood cell count; RC, regression coefficient; SFA, saturated fatty acid; WBC, white blood cell count; γ-GTP, γ-glutamyl transpeptidase

**Supplemental Table 9**. Multivariate panel data analysis for HR and vascular aging–related covariate trajectory

|  | FAS (*n* = 686) [Measurements = 3,341] | | | | | | | BCC (*n* = 678) [Measurements = 2,936] | | | | | | | CC (*n* = 648) [Measurements = 2,633] | | | | | | |
| --- | --- | --- | --- | --- | --- | --- | --- | --- | --- | --- | --- | --- | --- | --- | --- | --- | --- | --- | --- | --- | --- |
|  | RC | 95% CI | | | | | z score | RC | 95% CI | | | | | z score | RC | 95% CI | | | | | z score |
| Variables | *R^2^* = 0.137 | | | | | | | *R^2^* = 0.143 | | | | | | | *R^2^* = 0.143 | | | | | | |
| Age [1-year increment] | -0.008 | ( | -0.070 | – | 0.055 | ) | -0.24 | 0.008 | ( | -0.057 | – | 0.072 | ) | 0.23 | 0.018 | ( | -0.050 | – | 0.086 | ) | 0.52 |
| Female sex | 3.272 | ( | 0.990 | – | 5.554 | ) | **2.81** | 2.809 | ( | 0.450 | – | 5.169 | ) | **2.33** | 1.954 | ( | -0.507 | – | 4.415 | ) | 1.56 |
| Local area | 6.258 | ( | 4.757 | – | 7.759 | ) | **8.17** | 7.534 | ( | 5.965 | – | 9.103 | ) | **9.41** | 6.938 | ( | 5.315 | – | 8.560 | ) | **8.38** |
| BMI [1 kg/m^2^ increment] | -0.114 | ( | -0.339 | – | 0.110 | ) | -1 | -0.137 | ( | -0.372 | – | 0.097 | ) | -1.15 | -0.159 | ( | -0.403 | – | 0.085 | ) | -1.28 |
| Waist/hip ratio [0.1-point increment] | 0.617 | ( | -0.126 | – | 1.360 | ) | 1.63 | 0.683 | ( | -0.084 | – | 1.450 | ) | 1.74 | 0.724 | ( | -0.077 | – | 1.525 | ) | 1.77 |
| Smoker | -0.453 | ( | -1.574 | – | 0.669 | ) | -0.79 | -0.542 | ( | -1.730 | – | 0.647 | ) | -0.89 | -0.375 | ( | -1.724 | – | 0.974 | ) | -0.54 |
| FH of HD | 0.177 | ( | -0.474 | – | 0.829 | ) | 0.53 | -0.251 | ( | -0.975 | – | 0.472 | ) | -0.68 | -0.424 | ( | -1.201 | – | 0.353 | ) | -1.07 |
| RBC [10 count 10^12^/L increment] | 0.131 | ( | -0.050 | – | 0.312 | ) | 1.42 | 0.050 | ( | -0.139 | – | 0.238 | ) | 0.52 | 0.135 | ( | -0.062 | – | 0.331 | ) | 1.34 |
| WBC [10 count 10^9^/L increment] | 0.005 | ( | 0.002 | – | 0.008 | ) | **3.26** | 0.005 | ( | 0.002 | – | 0.008 | ) | **2.94** | 0.005 | ( | 0.002 | – | 0.008 | ) | **2.93** |
| PLT [1 count 10^9^/L increment] | 0.070 | ( | 0.016 | – | 0.124 | ) | **2.56** | 0.072 | ( | -0.015 | – | 0.158 | ) | 1.62 | 0.062 | ( | -0.029 | – | 0.152 | ) | 1.33 |
| Hemoglobin [1 mg/dL increment] | 0.064 | ( | -0.440 | – | 0.569 | ) | 0.25 | -0.041 | ( | -0.576 | – | 0.494 | ) | -0.15 | -0.378 | ( | -0.936 | – | 0.181 | ) | -1.33 |
| HDL-C [1 mg/dL increment] | -0.046 | ( | -0.076 | – | -0.017 | ) | **-3.07** | -0.038 | ( | -0.069 | – | -0.008 | ) | **-2.47** | -0.041 | ( | -0.073 | – | -0.009 | ) | **-2.5** |
| LDL-C [1 mg/dL increment] | -0.022 | ( | -0.034 | – | -0.009 | ) | **-3.27** | -0.019 | ( | -0.033 | – | -0.006 | ) | **-2.8** | -0.019 | ( | -0.033 | – | -0.004 | ) | **-2.52** |
| Triglycerides [1 mg/dL increment] | 0.003 | ( | -0.002 | – | 0.009 | ) | 1.18 | 0.003 | ( | -0.003 | – | 0.009 | ) | 1.08 | 0.001 | ( | -0.007 | – | 0.008 | ) | 0.21 |
| HbA1c [1 % increment] | -0.688 | ( | -1.629 | – | 0.253 | ) | -1.43 | -0.719 | ( | -1.702 | – | 0.264 | ) | -1.43 | -0.887 | ( | -1.962 | – | 0.188 | ) | -1.62 |
| HOMA-IR [1-point increment] | 0.795 | ( | 0.403 | – | 1.187 | ) | **3.97** | 1.164 | ( | 0.672 | – | 1.657 | ) | **4.63** | 1.413 | ( | 0.863 | – | 1.964 | ) | **5.03** |
| AST [1 IU/L increment] | -0.017 | ( | -0.087 | – | 0.053 | ) | -0.48 | 0.007 | ( | -0.065 | – | 0.079 | ) | 0.19 | 0.006 | ( | -0.068 | – | 0.080 | ) | 0.15 |
| ALT [1 IU/L increment] | 0.024 | ( | -0.026 | – | 0.073 | ) | 0.94 | 0.010 | ( | -0.041 | – | 0.061 | ) | 0.38 | 0.010 | ( | -0.043 | – | 0.062 | ) | 0.35 |
| γ-GTP [1 IU/L increment] | 0.012 | ( | -0.003 | – | 0.028 | ) | 1.57 | 0.011 | ( | -0.005 | – | 0.028 | ) | 1.31 | 0.014 | ( | -0.004 | – | 0.032 | ) | 1.51 |
| Comorbidity score [1-point increment] | 0.199 | ( | -0.262 | – | 0.659 | ) | 0.85 | 0.330 | ( | -0.166 | – | 0.826 | ) | 1.31 | 0.197 | ( | -0.350 | – | 0.745 | ) | 0.71 |
| Good sleep status | 0.436 | ( | -0.285 | – | 1.157 | ) | 1.19 | 0.489 | ( | -0.259 | – | 1.238 | ) | 1.28 | 0.571 | ( | -0.226 | – | 1.368 | ) | 1.4 |
| Hand grip strength [1 kg increment] | 0.052 | ( | -0.047 | – | 0.152 | ) | 1.03 | 0.030 | ( | -0.072 | – | 0.132 | ) | 0.58 | 0.004 | ( | -0.103 | – | 0.111 | ) | 0.07 |
| Leg power [10 w increment] | -0.002 | ( | -0.020 | – | 0.016 | ) | -0.19 | 0.007 | ( | -0.011 | – | 0.025 | ) | 0.73 | 0.004 | ( | -0.016 | – | 0.024 | ) | 0.38 |
| Seated forward bend [1 cm increment] | -0.096 | ( | -0.142 | – | -0.050 | ) | **-4.07** | -0.101 | ( | -0.149 | – | -0.053 | ) | **-4.11** | -0.087 | ( | -0.138 | – | -0.036 | ) | **-3.32** |
| Step counts [1000 steps/day increment] | -0.223 | ( | -0.335 | – | -0.112 | ) | **-3.92** | -0.244 | ( | -0.359 | – | -0.129 | ) | **-4.15** | -0.223 | ( | -0.344 | – | -0.103 | ) | **-3.64** |
| n-3/n-6 FA ratio [1-point increment] | 1.482 | ( | -3.636 | – | 6.601 | ) | 0.57 | 0.570 | ( | -4.914 | – | 6.054 | ) | 0.2 | 0.583 | ( | -5.194 | – | 6.360 | ) | 0.2 |
| SFA intake [1 % energy/day increment] | -0.036 | ( | -0.245 | – | 0.173 | ) | -0.34 | -0.042 | ( | -0.258 | – | 0.173 | ) | -0.38 | -0.022 | ( | -0.250 | – | 0.207 | ) | -0.19 |
| Alcohol consumption [1 % energy/day increment] | -0.017 | ( | -0.092 | – | 0.057 | ) | -0.46 | -0.038 | ( | -0.115 | – | 0.038 | ) | -0.98 | -0.029 | ( | -0.109 | – | 0.051 | ) | -0.71 |
| Salt intake [1 g/1,000 kcal/day increment] | -0.047 | ( | -0.357 | – | 0.264 | ) | -0.29 | -0.005 | ( | -0.326 | – | 0.316 | ) | -0.03 | -0.020 | ( | -0.362 | – | 0.321 | ) | -0.12 |
| Sugar intake [1 % energy/day increment] | -0.002 | ( | -0.219 | – | 0.215 | ) | -0.02 | 0.022 | ( | -0.203 | – | 0.247 | ) | 0.19 | -0.022 | ( | -0.258 | – | 0.215 | ) | -0.18 |
| Meat intake [10 g/1,000 kcal/day increment] | 0.228 | ( | 0.031 | – | 0.425 | ) | **2.27** | 0.257 | ( | 0.056 | – | 0.459 | ) | **2.5** | 0.264 | ( | 0.051 | – | 0.477 | ) | **2.43** |
| FV intake [10 g/1,000 kcal/day increment] | 0.017 | ( | -0.019 | – | 0.053 | ) | 0.91 | 0.020 | ( | -0.017 | – | 0.057 | ) | 1.06 | 0.028 | ( | -0.012 | – | 0.067 | ) | 1.37 |
| Pulses intake [10 g/1,000 kcal/day increment] | -0.059 | ( | -0.204 | – | 0.085 | ) | -0.81 | -0.094 | ( | -0.242 | – | 0.055 | ) | -1.24 | -0.141 | ( | -0.299 | – | 0.018 | ) | -1.74 |

The values are shown as regression coefficients and 95% confidence intervals for the effect of HR per unit increment of each covariate. The z scores indicated in bold are statistically significant (≤-1.96 or ≥1.96). Sex, area, and FH of HD were time-stable variables, while the other covariates were time-varying variables. ALT, alanine aminotransferase; AST, aspartate aminotransferase; BCC, biological vascular aging indicator complete case; BMI, body mass index; CC, complete case; FA, fatty acid; FAS, full analysis set; FH, family history; FV, fruits and vegetables; HbA1c, hemoglobin A1C; HD, heart disease; HDL-C, high density lipoprotein cholesterol; HOMA-IR, homeostasis model assessment of insulin resistance; HR, heart rate; LDL-C, low density lipoprotein cholesterol; PLT, platelets; RBC, red blood cell count; RC, regression coefficient; SFA, saturated fatty acid; WBC, white blood cell count; γ-GTP, γ-glutamyl transpeptidase

**Supplemental Table 10**. Multivariate panel data analysis for DD and vascular aging–related covariate trajectory

|  | FAS (*n* = 681) [Measurements = 3,003] | | | | | | | BCC (*n* = 678) [Measurements = 2,936] | | | | | | | CC (*n* = 648) [Measurements = 2,633] | | | | | | |
| --- | --- | --- | --- | --- | --- | --- | --- | --- | --- | --- | --- | --- | --- | --- | --- | --- | --- | --- | --- | --- | --- |
|  | RC | 95% CI | | | | | z score | RC | 95% CI | | | | | z score | RC | 95% CI | | | | | z score |
| Variables | *R^2^* = 0.304 | | | | | | | *R^2^* = 0.308 | | | | | | | *R^2^* = 0.306 | | | | | | |
| Age [1-year increment] | 0.275 | ( | 0.244 | – | 0.306 | ) | **17.38** | 0.265 | ( | 0.234 | – | 0.297 | ) | **16.59** | 0.268 | ( | 0.235 | – | 0.301 | ) | **15.8** |
| Female sex | -5.760 | ( | -6.987 | – | -4.533 | ) | **-9.2** | -5.873 | ( | -7.106 | – | -4.641 | ) | **-9.34** | -5.538 | ( | -6.820 | – | -4.255 | ) | **-8.46** |
| Local area | 0.024 | ( | -0.944 | – | 0.992 | ) | 0.05 | -0.059 | ( | -1.030 | – | 0.911 | ) | -0.12 | 0.052 | ( | -0.939 | – | 1.043 | ) | 0.1 |
| BMI [1 kg/m^2^ increment] | 0.303 | ( | 0.189 | – | 0.418 | ) | **5.19** | 0.330 | ( | 0.214 | – | 0.447 | ) | **5.56** | 0.328 | ( | 0.205 | – | 0.450 | ) | **5.25** |
| Waist/hip ratio [0.1-point increment] | 0.047 | ( | -0.249 | – | 0.342 | ) | 0.31 | 0.011 | ( | -0.288 | – | 0.311 | ) | 0.07 | -0.002 | ( | -0.318 | – | 0.314 | ) | -0.01 |
| Smoker | 0.692 | ( | 0.165 | – | 1.220 | ) | **2.57** | 0.716 | ( | 0.184 | – | 1.248 | ) | **2.64** | 1.121 | ( | 0.451 | – | 1.791 | ) | **3.28** |
| FH of HD | -0.071 | ( | -0.342 | – | 0.200 | ) | -0.51 | -0.065 | ( | -0.342 | – | 0.211 | ) | -0.46 | -0.041 | ( | -0.341 | – | 0.259 | ) | -0.27 |
| RBC [10 count 10^12^/L increment] | -0.044 | ( | -0.122 | – | 0.034 | ) | -1.1 | -0.032 | ( | -0.112 | – | 0.048 | ) | -0.79 | -0.047 | ( | -0.130 | – | 0.037 | ) | -1.09 |
| WBC [10 count 10^9^/L increment] | 0.000 | ( | -0.001 | – | 0.001 | ) | -0.01 | 0.000 | ( | -0.001 | – | 0.001 | ) | -0.01 | 0.000 | ( | -0.001 | – | 0.001 | ) | -0.06 |
| PLT [1 count 10^9^/L increment] | -0.007 | ( | -0.043 | – | 0.029 | ) | -0.38 | -0.003 | ( | -0.039 | – | 0.032 | ) | -0.19 | -0.002 | ( | -0.040 | – | 0.036 | ) | -0.12 |
| Hemoglobin [1 mg/dL increment] | -0.079 | ( | -0.288 | – | 0.130 | ) | -0.74 | -0.143 | ( | -0.359 | – | 0.074 | ) | -1.29 | -0.101 | ( | -0.327 | – | 0.126 | ) | -0.87 |
| HDL-C [1 mg/dL increment] | 0.005 | ( | -0.008 | – | 0.018 | ) | 0.75 | 0.007 | ( | -0.006 | – | 0.021 | ) | 1.11 | 0.007 | ( | -0.007 | – | 0.021 | ) | 1.03 |
| LDL-C [1 mg/dL increment] | -0.005 | ( | -0.010 | – | 0.000 | ) | -1.87 | -0.005 | ( | -0.010 | – | 0.000 | ) | -1.86 | -0.006 | ( | -0.012 | – | -0.001 | ) | **-2.15** |
| Triglycerides [1 mg/dL increment] | 0.001 | ( | -0.001 | – | 0.003 | ) | 0.96 | 0.001 | ( | -0.001 | – | 0.004 | ) | 1.32 | 0.003 | ( | 0.000 | – | 0.006 | ) | 1.96 |
| HbA1c [1 % increment] | 0.126 | ( | -0.261 | – | 0.512 | ) | 0.64 | 0.137 | ( | -0.255 | – | 0.529 | ) | 0.69 | -0.009 | ( | -0.443 | – | 0.425 | ) | -0.04 |
| HOMA-IR [1-point increment] | 0.011 | ( | -0.159 | – | 0.180 | ) | 0.12 | 0.076 | ( | -0.109 | – | 0.261 | ) | 0.8 | 0.062 | ( | -0.147 | – | 0.272 | ) | 0.58 |
| AST [1 IU/L increment] | 0.035 | ( | 0.008 | – | 0.063 | ) | **2.57** | 0.038 | ( | 0.011 | – | 0.065 | ) | **2.73** | 0.036 | ( | 0.008 | – | 0.064 | ) | **2.51** |
| ALT [1 IU/L increment] | -0.024 | ( | -0.043 | – | -0.004 | ) | **-2.38** | -0.025 | ( | -0.045 | – | -0.006 | ) | **-2.52** | -0.026 | ( | -0.046 | – | -0.006 | ) | **-2.5** |
| γ-GTP [1 IU/L increment] | 0.001 | ( | -0.005 | – | 0.007 | ) | 0.31 | 0.000 | ( | -0.006 | – | 0.007 | ) | 0.12 | 0.002 | ( | -0.006 | – | 0.010 | ) | 0.47 |
| Comorbidity score [1-point increment] | 0.078 | ( | -0.108 | – | 0.265 | ) | 0.83 | 0.059 | ( | -0.131 | – | 0.248 | ) | 0.6 | 0.044 | ( | -0.167 | – | 0.255 | ) | 0.41 |
| Good sleep status | 0.033 | ( | -0.243 | – | 0.309 | ) | 0.23 | 0.012 | ( | -0.268 | – | 0.292 | ) | 0.08 | 0.102 | ( | -0.199 | – | 0.403 | ) | 0.66 |
| Hand grip strength [1 kg increment] | 0.014 | ( | -0.028 | – | 0.056 | ) | 0.65 | 0.012 | ( | -0.031 | – | 0.054 | ) | 0.54 | 0.018 | ( | -0.027 | – | 0.063 | ) | 0.8 |
| Leg power [10 w increment] | -0.001 | ( | -0.008 | – | 0.006 | ) | -0.36 | -0.002 | ( | -0.009 | – | 0.005 | ) | -0.62 | -0.002 | ( | -0.010 | – | 0.005 | ) | -0.62 |
| Seated forward bend [1 cm increment] | -0.016 | ( | -0.035 | – | 0.004 | ) | -1.59 | -0.018 | ( | -0.038 | – | 0.002 | ) | -1.79 | -0.020 | ( | -0.041 | – | 0.001 | ) | -1.86 |
| Step counts [1000 steps/day increment] | -0.059 | ( | -0.104 | – | -0.014 | ) | **-2.55** | -0.055 | ( | -0.100 | – | -0.009 | ) | **-2.35** | -0.043 | ( | -0.005 | – | -0.091 | ) | **-1.97** |
| n-3/n-6 FA ratio [1-point increment] | -0.259 | ( | -2.173 | – | 1.656 | ) | -0.26 | 0.651 | ( | -1.367 | – | 2.670 | ) | 0.63 | 1.218 | ( | -0.922 | – | 3.358 | ) | 1.12 |
| SFA intake [1 % energy/day increment] | 0.031 | ( | -0.048 | – | 0.109 | ) | 0.76 | 0.028 | ( | -0.052 | – | 0.108 | ) | 0.68 | 0.037 | ( | -0.049 | – | 0.122 | ) | 0.84 |
| Alcohol consumption [1 % energy/day increment] | 0.012 | ( | -0.018 | – | 0.043 | ) | 0.81 | 0.014 | ( | -0.017 | – | 0.044 | ) | 0.88 | 0.013 | ( | -0.020 | – | 0.045 | ) | 0.77 |
| Salt intake [1 g/1,000 kcal/day increment] | 0.038 | ( | -0.079 | – | 0.156 | ) | 0.64 | 0.026 | ( | -0.094 | – | 0.145 | ) | 0.42 | -0.028 | ( | -0.156 | – | 0.099 | ) | -0.44 |
| Sugar intake [1 % energy/day increment] | 0.014 | ( | -0.071 | – | 0.098 | ) | 0.32 | 0.014 | ( | -0.072 | – | 0.099 | ) | 0.31 | 0.019 | ( | -0.071 | – | 0.109 | ) | 0.41 |
| Meat intake [10 g/1,000 kcal/day increment] | 0.105 | ( | 0.031 | – | 0.179 | ) | **2.77** | 0.104 | ( | 0.029 | – | 0.179 | ) | **2.71** | 0.132 | ( | 0.052 | – | 0.212 | ) | **3.25** |
| FV intake [10 g/1,000 kcal/day increment] | -0.003 | ( | -0.017 | – | 0.011 | ) | -0.38 | -0.001 | ( | -0.015 | – | 0.013 | ) | -0.18 | 0.006 | ( | -0.009 | – | 0.021 | ) | 0.76 |
| Pulses intake [10 g/1,000 kcal/day increment] | -0.031 | ( | -0.086 | – | 0.024 | ) | -1.12 | -0.029 | ( | -0.084 | – | 0.026 | ) | -1.03 | -0.037 | ( | -0.096 | – | 0.022 | ) | -1.24 |

The values are shown as regression coefficients and 95% confidence intervals for the effect of DD per unit increment of each covariate. The regression coefficients and 95% confidence intervals are shown in the estimated value corrected by 10 because the estimated value was small. The z scores indicated in bold are statistically significant (≤-1.96 or ≥1.96). Sex, area, and FH of HD were time-stable variables, while the other covariates were time-varying variables. ALT, alanine aminotransferase; AST, aspartate aminotransferase; BCC, biological vascular aging indicator complete case; BMI, body mass index; CC, complete case; DD, common carotid diastolic diameter, FA, fatty acid; FAS, full analysis set; FH, family history; FV, fruits and vegetables; HbA1c, hemoglobin A1C; HD, heart disease; HDL-C, high density lipoprotein cholesterol; HOMA-IR, homeostasis model assessment of insulin resistance; LDL-C, low density lipoprotein cholesterol; PLT, platelets; RBC, red blood cell count; RC, regression coefficient; SFA, saturated fatty acid; WBC, white blood cell count; γ-GTP, γ-glutamyl transpeptidase

**Supplemental Table 11**. Multivariate panel data analysis for MBV and vascular aging–related covariate trajectory

|  | FAS (*n* = 686) [Measurements = 3,161] | | | | | | | BCC (*n* = 678) [Measurements = 2,936] | | | | | | | CC (*n* = 648) [Measurements = 2,633] | | | | | | |
| --- | --- | --- | --- | --- | --- | --- | --- | --- | --- | --- | --- | --- | --- | --- | --- | --- | --- | --- | --- | --- | --- |
|  | RC | 95% CI | | | | | z score | RC | 95% CI | | | | | z score | RC | 95% CI | | | | | z score |
| Variables | *R^2^* = 0.282 | | | | | | | *R^2^* = 0.261 | | | | | | | *R^2^* = 0.263 | | | | | | |
| Age [1-year increment] | -0.100 | ( | -0.141 | – | -0.059 | ) | **-4.79** | -0.095 | ( | -0.138 | – | -0.053 | ) | **-4.4** | -0.081 | ( | -0.126 | – | -0.036 | ) | **-3.53** |
| Female sex | -0.954 | ( | -2.449 | – | 0.540 | ) | -1.25 | -1.017 | ( | -2.554 | – | 0.521 | ) | -1.3 | -0.900 | ( | -2.515 | – | 0.715 | ) | -1.09 |
| Local area | -7.936 | ( | -8.882 | – | -6.991 | ) | **-16.46** | -7.771 | ( | -8.753 | – | -6.790 | ) | **-15.52** | -7.789 | ( | -8.818 | – | -6.760 | ) | **-14.83** |
| BMI [1 kg/m^2^ increment] | 0.011 | ( | -0.135 | – | 0.157 | ) | 0.15 | 0.047 | ( | -0.105 | – | 0.198 | ) | 0.6 | 0.030 | ( | -0.129 | – | 0.189 | ) | 0.37 |
| Waist/hip ratio [0.1-point increment] | -0.496 | ( | -1.016 | – | 0.024 | ) | -1.87 | -0.545 | ( | -1.087 | – | -0.003 | ) | **-1.97** | -0.595 | ( | -1.163 | – | -0.028 | ) | **-2.06** |
| Smoker | -0.469 | ( | -1.238 | – | 0.300 | ) | -1.2 | -0.420 | ( | -1.205 | – | 0.364 | ) | -1.05 | -0.597 | ( | -1.468 | – | 0.273 | ) | -1.35 |
| FH of HD | -0.173 | ( | -0.641 | – | 0.295 | ) | -0.72 | -0.154 | ( | -0.669 | – | 0.361 | ) | -0.59 | -0.266 | ( | -0.819 | – | 0.287 | ) | -0.94 |
| RBC [10 count 10^12^/L increment] | 0.081 | ( | -0.041 | – | 0.204 | ) | 1.3 | 0.047 | ( | -0.081 | – | 0.174 | ) | 0.72 | 0.072 | ( | -0.062 | – | 0.206 | ) | 1.05 |
| WBC [10 count 10^9^/L increment] | 0.002 | ( | 0.000 | – | 0.004 | ) | 1.65 | 0.002 | ( | 0.000 | – | 0.004 | ) | 1.61 | 0.002 | ( | 0.000 | – | 0.005 | ) | **2.09** |
| PLT [1 count 10^9^/L increment] | -0.004 | ( | -0.041 | – | 0.034 | ) | -0.19 | 0.029 | ( | -0.030 | – | 0.089 | ) | 0.98 | 0.021 | ( | -0.041 | – | 0.084 | ) | 0.67 |
| Hemoglobin [1 mg/dL increment] | -0.812 | ( | -1.158 | – | -0.466 | ) | **-4.6** | -0.718 | ( | -1.088 | – | -0.347 | ) | **-3.8** | -0.847 | ( | -1.236 | – | -0.457 | ) | **-4.26** |
| HDL-C [1 mg/dL increment] | -0.002 | ( | -0.022 | – | 0.018 | ) | -0.18 | -0.003 | ( | -0.023 | – | 0.018 | ) | -0.28 | 0.000 | ( | -0.021 | – | 0.022 | ) | 0.01 |
| LDL-C [1 mg/dL increment] | 0.007 | ( | -0.002 | – | 0.016 | ) | 1.56 | 0.008 | ( | -0.001 | – | 0.018 | ) | 1.72 | 0.007 | ( | -0.003 | – | 0.017 | ) | 1.44 |
| Triglycerides [1 mg/dL increment] | -0.002 | ( | -0.006 | – | 0.002 | ) | -1.19 | -0.003 | ( | -0.007 | – | 0.001 | ) | -1.44 | -0.002 | ( | -0.008 | – | 0.003 | ) | -0.89 |
| HbA1c [1 % increment] | -0.513 | ( | -1.179 | – | 0.153 | ) | -1.51 | -0.479 | ( | -1.163 | – | 0.206 | ) | -1.37 | -0.135 | ( | -0.885 | – | 0.614 | ) | -0.35 |
| HOMA-IR [1-point increment] | -0.270 | ( | -0.611 | – | 0.070 | ) | -1.56 | -0.313 | ( | -0.669 | – | 0.043 | ) | -1.73 | -0.392 | ( | -0.789 | – | 0.005 | ) | -1.93 |
| AST [1 IU/L increment] | 0.013 | ( | -0.036 | – | 0.062 | ) | 0.52 | 0.020 | ( | -0.031 | – | 0.072 | ) | 0.78 | 0.014 | ( | -0.039 | – | 0.067 | ) | 0.51 |
| ALT [1 IU/L increment] | -0.008 | ( | -0.043 | – | 0.026 | ) | -0.47 | -0.014 | ( | -0.051 | – | 0.022 | ) | -0.76 | -0.017 | ( | -0.055 | – | 0.021 | ) | -0.89 |
| γ-GTP [1 IU/L increment] | -0.001 | ( | -0.012 | – | 0.009 | ) | -0.21 | 0.000 | ( | -0.011 | – | 0.011 | ) | 0.03 | 0.000 | ( | -0.012 | – | 0.012 | ) | 0.05 |
| Comorbidity score [1-point increment] | -0.380 | ( | -0.717 | – | -0.043 | ) | **-2.21** | -0.428 | ( | -0.781 | – | -0.074 | ) | **-2.37** | -0.488 | ( | -0.880 | – | -0.097 | ) | **-2.44** |
| Good sleep status | -0.351 | ( | -0.866 | – | 0.163 | ) | -1.34 | -0.229 | ( | -0.769 | – | 0.311 | ) | -0.83 | -0.550 | ( | -1.124 | – | 0.025 | ) | -1.88 |
| Hand grip strength [1 kg increment] | -0.119 | ( | -0.187 | – | -0.051 | ) | **-3.42** | -0.118 | ( | -0.188 | – | -0.048 | ) | **-3.3** | -0.111 | ( | -0.184 | – | -0.038 | ) | **-2.96** |
| Leg power [10 w increment] | 0.012 | ( | 0.000 | – | 0.025 | ) | 1.91 | 0.011 | ( | -0.002 | – | 0.024 | ) | 1.68 | 0.015 | ( | 0.001 | – | 0.029 | ) | 2.14 |
| Seated forward bend [1 cm increment] | 0.015 | ( | -0.017 | – | 0.046 | ) | 0.91 | 0.017 | ( | -0.016 | – | 0.050 | ) | 1.02 | 0.018 | ( | -0.017 | – | 0.053 | ) | 1.01 |
| Step counts [1000 steps/day increment] | -0.023 | ( | -0.100 | – | 0.054 | ) | -0.58 | -0.026 | ( | -0.106 | – | 0.055 | ) | -0.62 | -0.029 | ( | -0.113 | – | 0.055 | ) | -0.67 |
| n-3/n-6 FA ratio [1-point increment] | -0.436 | ( | -4.159 | – | 3.287 | ) | -0.23 | -1.178 | ( | -5.181 | – | 2.825 | ) | -0.58 | -1.623 | ( | -5.843 | – | 2.597 | ) | -0.75 |
| SFA intake [1 % energy/day increment] | -0.024 | ( | -0.173 | – | 0.126 | ) | -0.31 | 0.008 | ( | -0.149 | – | 0.164 | ) | 0.1 | -0.010 | ( | -0.176 | – | 0.157 | ) | -0.11 |
| Alcohol consumption [1 % energy/day increment] | -0.060 | ( | -0.112 | – | -0.008 | ) | **-2.28** | -0.057 | ( | -0.111 | – | -0.004 | ) | **-2.1** | -0.056 | ( | -0.112 | – | 0.000 | ) | **-1.96** |
| Salt intake [1 g/1,000 kcal/day increment] | -0.206 | ( | -0.018 | – | -0.429 | ) | **-1.98** | -0.187 | ( | -0.421 | – | 0.046 | ) | -1.57 | -0.251 | ( | -0.500 | – | -0.003 | ) | **-1.98** |
| Sugar intake [1 % energy/day increment] | -0.028 | ( | -0.181 | – | 0.126 | ) | -0.35 | -0.014 | ( | -0.174 | – | 0.147 | ) | -0.17 | -0.024 | ( | -0.194 | – | 0.145 | ) | -0.28 |
| Meat intake [10 g/1,000 kcal/day increment] | 0.105 | ( | -0.036 | – | 0.246 | ) | 1.46 | 0.096 | ( | -0.050 | – | 0.243 | ) | 1.29 | 0.084 | ( | -0.071 | – | 0.239 | ) | 1.06 |
| FV intake [10 g/1,000 kcal/day increment] | 0.005 | ( | -0.021 | – | 0.031 | ) | 0.38 | 0.009 | ( | -0.018 | – | 0.036 | ) | 0.66 | 0.006 | ( | -0.022 | – | 0.034 | ) | 0.41 |
| Pulses intake [10 g/1,000 kcal/day increment] | -0.028 | ( | -0.132 | – | 0.076 | ) | -0.53 | -0.056 | ( | -0.164 | – | 0.052 | ) | -1.01 | -0.047 | ( | -0.162 | – | 0.068 | ) | -0.8 |

The values are shown as regression coefficients and 95% confidence intervals for the effect of MBV per unit increment of each covariate. The z scores indicated in bold are statistically significant (≤-1.96 or ≥1.96). Sex, area, and FH of HD were time-stable variables, while the other covariates were time-varying variables. ALT, alanine aminotransferase; AST, aspartate aminotransferase; BCC, biological vascular aging indicator complete case; BMI, body mass index; CC, complete case; FA, fatty acid; FAS, full analysis set; FH, family history; FV, fruits and vegetables; HbA1c, hemoglobin A1C; HD, heart disease; HDL-C, high density lipoprotein cholesterol; HOMA-IR, homeostasis model assessment of insulin resistance; LDL-C, low density lipoprotein cholesterol; MBV, carotid artery mean blood velocity; PLT, platelets; RBC, red blood cell count; RC, regression coefficient; SFA, saturated fatty acid; WBC, white blood cell count; γ-GTP, γ-glutamyl transpeptidase

**Supplemental Table 12**. Multivariate panel data analysis for BF and vascular aging–related covariate trajectory

|  | FAS (*n* = 681) [Measurements = 2,995] | | | | | | | BCC (*n* = 678) [Measurements = 2,936] | | | | | | | CC (*n* = 648) [Measurements = 2,633] | | | | | | |
| --- | --- | --- | --- | --- | --- | --- | --- | --- | --- | --- | --- | --- | --- | --- | --- | --- | --- | --- | --- | --- | --- |
|  | RC | 95% CI | | | | | z score | RC | 95% CI | | | | | z score | RC | 95% CI | | | | | z score |
| Variables | *R^2^* = 0.227 | | | | | | | *R^2^* = 0.229 | | | | | | | *R^2^* = 0.244 | | | | | | |
| Age [1-year increment] | 1.748 | ( | 0.921 | – | 2.574 | ) | **4.14** | 1.730 | ( | 0.896 | – | 2.564 | ) | **4.07** | 2.025 | ( | 1.146 | – | 2.904 | ) | **4.51** |
| Female sex | -84.323 | ( | -114.404 | – | -54.241 | ) | **-5.49** | -86.874 | ( | -117.136 | – | -56.611 | ) | **-5.63** | -80.166 | ( | -111.660 | – | -48.672 | ) | **-4.99** |
| Local area | -141.863 | ( | -161.071 | – | -122.655 | ) | **-14.48** | -141.721 | ( | -161.064 | – | -122.378 | ) | **-14.36** | -140.234 | ( | -160.289 | – | -120.180 | ) | **-13.71** |
| BMI [1 kg/m^2^ increment] | 5.905 | ( | 2.945 | – | 8.864 | ) | **3.91** | 6.150 | ( | 3.170 | – | 9.130 | ) | **4.05** | 5.467 | ( | 2.372 | – | 8.563 | ) | **3.46** |
| Waist/hip ratio [0.1-point increment] | -8.050 | ( | -18.581 | – | 2.482 | ) | -1.5 | -8.676 | ( | -19.324 | – | 1.972 | ) | -1.6 | -9.624 | ( | -20.712 | – | 1.464 | ) | -1.7 |
| Smoker | 5.960 | ( | -9.375 | – | 21.295 | ) | 0.76 | 7.393 | ( | -8.048 | – | 22.834 | ) | 0.94 | 8.377 | ( | -8.580 | – | 25.333 | ) | 0.97 |
| FH of HD | -5.906 | ( | -15.847 | – | 4.034 | ) | -1.16 | -4.595 | ( | -14.704 | – | 5.513 | ) | -0.89 | -6.147 | ( | -16.952 | – | 4.659 | ) | -1.11 |
| RBC [10 count 10^12^/L increment] | 0.369 | ( | -2.105 | – | 2.844 | ) | 0.29 | 0.398 | ( | -2.109 | – | 2.906 | ) | 0.31 | 0.894 | ( | -1.716 | – | 3.504 | ) | 0.67 |
| WBC [10 count 10^9^/L increment] | 0.028 | ( | -0.015 | – | 0.071 | ) | 1.26 | 0.028 | ( | -0.015 | – | 0.072 | ) | 1.27 | 0.039 | ( | -0.007 | – | 0.084 | ) | 1.67 |
| PLT [1 count 10^9^/L increment] | 0.555 | ( | -0.598 | – | 1.708 | ) | 0.94 | 0.540 | ( | -0.621 | – | 1.702 | ) | 0.91 | 0.389 | ( | -0.824 | – | 1.602 | ) | 0.63 |
| Hemoglobin [1 mg/dL increment] | -14.452 | ( | -21.550 | – | -7.354 | ) | **-3.99** | -15.534 | ( | -22.805 | – | -8.262 | ) | **-4.19** | -18.104 | ( | -25.705 | – | -10.504 | ) | **-4.67** |
| HDL-C [1 mg/dL increment] | -0.019 | ( | -0.418 | – | 0.381 | ) | -0.09 | 0.011 | ( | -0.392 | – | 0.414 | ) | 0.05 | 0.056 | ( | -0.365 | – | 0.477 | ) | 0.26 |
| LDL-C [1 mg/dL increment] | 0.034 | ( | -0.149 | – | 0.217 | ) | 0.37 | 0.033 | ( | -0.152 | – | 0.217 | ) | 0.35 | -0.031 | ( | -0.227 | – | 0.166 | ) | -0.3 |
| Triglycerides [1 mg/dL increment] | -0.039 | ( | -0.119 | – | 0.042 | ) | -0.93 | -0.036 | ( | -0.117 | – | 0.045 | ) | -0.87 | 0.001 | ( | -0.101 | – | 0.103 | ) | 0.02 |
| HbA1c [1 % increment] | -1.460 | ( | -14.781 | – | 11.860 | ) | -0.21 | -2.968 | ( | -16.414 | – | 10.478 | ) | -0.43 | 2.083 | ( | -12.549 | – | 16.716 | ) | 0.28 |
| HOMA-IR [1-point increment] | -3.727 | ( | -10.537 | – | 3.084 | ) | -1.07 | -2.910 | ( | -9.894 | – | 4.074 | ) | -0.82 | -4.093 | ( | -11.862 | – | 3.675 | ) | -1.03 |
| AST [1 IU/L increment] | 0.443 | ( | -0.558 | – | 1.444 | ) | 0.87 | 0.474 | ( | -0.533 | – | 1.480 | ) | 0.92 | 0.287 | ( | -0.751 | – | 1.325 | ) | 0.54 |
| ALT [1 IU/L increment] | -0.282 | ( | -0.992 | – | 0.428 | ) | -0.78 | -0.285 | ( | -1.000 | – | 0.430 | ) | -0.78 | -0.337 | ( | -1.073 | – | 0.398 | ) | -0.9 |
| γ-GTP [1 IU/L increment] | 0.023 | ( | -0.188 | – | 0.234 | ) | 0.21 | 0.038 | ( | -0.183 | – | 0.258 | ) | 0.34 | 0.058 | ( | -0.175 | – | 0.291 | ) | 0.49 |
| Comorbidity score [1-point increment] | 2.128 | ( | -4.718 | – | 8.973 | ) | 0.61 | 2.183 | ( | -4.758 | – | 9.124 | ) | 0.62 | 1.548 | ( | -6.106 | – | 9.202 | ) | 0.4 |
| Good sleep status | -4.040 | ( | -14.507 | – | 6.426 | ) | -0.76 | -3.969 | ( | -14.562 | – | 6.625 | ) | -0.73 | -8.307 | ( | -19.534 | – | 2.920 | ) | -1.45 |
| Hand grip strength [1 kg increment] | -1.363 | ( | -2.729 | – | 0.002 | ) | -1.96 | -1.342 | ( | -2.717 | – | 0.033 | ) | -1.91 | -1.140 | ( | -2.572 | – | 0.293 | ) | -1.56 |
| Leg power [10 w increment] | 0.232 | ( | -0.017 | – | 0.482 | ) | 1.82 | 0.204 | ( | -0.048 | – | 0.457 | ) | 1.59 | 0.296 | ( | 0.021 | – | 0.570 | ) | **2.11** |
| Seated forward bend [1 cm increment] | -0.052 | ( | -0.697 | – | 0.594 | ) | -0.16 | -0.117 | ( | -0.768 | – | 0.535 | ) | -0.35 | -0.158 | ( | -0.843 | – | 0.527 | ) | -0.45 |
| Step counts [1000 steps/day increment] | -1.516 | ( | -3.079 | – | 0.047 | ) | -1.9 | -1.378 | ( | -2.956 | – | 0.200 | ) | -1.71 | -1.306 | ( | -2.945 | – | 0.334 | ) | -1.56 |
| n-3/n-6 FA ratio [1-point increment] | -23.056 | ( | -97.790 | – | 51.678 | ) | -0.6 | -13.291 | ( | -91.798 | – | 65.217 | ) | -0.33 | -9.644 | ( | -92.154 | – | 72.865 | ) | -0.23 |
| SFA intake [1 % energy/day increment] | 0.421 | ( | -2.599 | – | 3.441 | ) | 0.27 | 0.299 | ( | -2.766 | – | 3.363 | ) | 0.19 | 0.381 | ( | -2.864 | – | 3.627 | ) | 0.23 |
| Alcohol consumption [1 % energy/day increment] | 0.062 | ( | -0.981 | – | 1.105 | ) | 0.12 | -0.008 | ( | -1.058 | – | 1.042 | ) | -0.01 | 0.021 | ( | -1.074 | – | 1.115 | ) | 0.04 |
| Salt intake [1 g/1,000 kcal/day increment] | -1.629 | ( | -6.140 | – | 2.882 | ) | -0.71 | -1.452 | ( | -6.029 | – | 3.124 | ) | -0.62 | -3.294 | ( | -8.151 | – | 1.564 | ) | -1.33 |
| Sugar intake [1 % energy/day increment] | 0.654 | ( | -2.464 | – | 3.771 | ) | 0.41 | 0.521 | ( | -2.636 | – | 3.679 | ) | 0.32 | 0.666 | ( | -2.648 | – | 3.980 | ) | 0.39 |
| Meat intake [10 g/1,000 kcal/day increment] | 4.038 | ( | 1.200 | – | 6.876 | ) | **2.79** | 3.936 | ( | 1.069 | – | 6.804 | ) | **2.69** | 4.027 | ( | 1.006 | – | 7.048 | ) | **2.61** |
| FV intake [10 g/1,000 kcal/day increment] | 0.099 | ( | -0.421 | – | 0.619 | ) | 0.37 | 0.145 | ( | -0.380 | – | 0.670 | ) | 0.54 | 0.137 | ( | -0.419 | – | 0.694 | ) | 0.48 |
| Pulses intake [10 g/1,000 kcal/day increment] | -1.437 | ( | -3.536 | – | 0.662 | ) | -1.34 | -1.586 | ( | -3.700 | – | 0.528 | ) | -1.47 | -1.563 | ( | -3.812 | – | 0.687 | ) | -1.36 |

The values are shown as regression coefficients and 95% confidence intervals for the effect of BF per unit increment of each covariate. The z scores indicated in bold are statistically significant (≤-1.96 or ≥1.96). Sex, area, and FH of HD were time-stable variables, while the other covariates were time-varying variables. ALT, alanine aminotransferase; AST, aspartate aminotransferase; BCC, biological vascular aging indicator complete case; BF, blood flow; BMI, body mass index; CC, complete case; FA, fatty acid; FAS, full analysis set; FH, family history; FV, fruits and vegetables; HbA1c, hemoglobin A1C; HD, heart disease; HDL-C, high density lipoprotein cholesterol; HOMA-IR, homeostasis model assessment of insulin resistance; LDL-C, low density lipoprotein cholesterol; PLT, platelets; RBC, red blood cell count; RC, regression coefficient; SFA, saturated fatty acid; WBC, white blood cell count; γ-GTP, γ-glutamyl transpeptidase

**Supplemental Table 13**. Multivariate panel data analysis for IMT and vascular aging–related covariate trajectory

|  | FAS (*n* = 681) [Measurements = 3,002] | | | | | | | BCC (*n* = 678) [Measurements = 2,936] | | | | | | | CC (*n* = 648) [Measurements = 2,633] | | | | | | |
| --- | --- | --- | --- | --- | --- | --- | --- | --- | --- | --- | --- | --- | --- | --- | --- | --- | --- | --- | --- | --- | --- |
|  | RC | 95% CI | | | | | z score | RC | 95% CI | | | | | z score | RC | 95% CI | | | | | z score |
| Variables | *R^2^* = 0.438 | | | | | | | *R^2^* = 0.440 | | | | | | | *R^2^* = 0.415 | | | | | | |
| Age [1-year increment] | 0.088 | ( | 0.081 | – | 0.095 | ) | **24.8** | 0.087 | ( | 0.080 | – | 0.094 | ) | **24.44** | 0.083 | ( | 0.076 | – | 0.090 | ) | **22.25** |
| Female sex | -0.054 | ( | -0.308 | – | 0.200 | ) | -0.42 | -0.049 | ( | -0.304 | – | 0.205 | ) | -0.38 | 0.001 | ( | -0.263 | – | 0.266 | ) | 0.01 |
| Local area | 0.050 | ( | -0.121 | – | 0.220 | ) | 0.57 | 0.035 | ( | -0.136 | – | 0.206 | ) | 0.4 | 0.024 | ( | -0.152 | – | 0.200 | ) | 0.27 |
| BMI [1 kg/m^2^ increment] | 0.043 | ( | 0.017 | – | 0.068 | ) | **3.31** | 0.047 | ( | 0.022 | – | 0.072 | ) | **3.63** | 0.053 | ( | 0.026 | – | 0.079 | ) | **3.92** |
| Waist/hip ratio [0.1-point increment] | -0.106 | ( | -0.187 | – | -0.025 | ) | **-2.57** | -0.140 | ( | -0.221 | – | -0.059 | ) | **-3.38** | -0.139 | ( | -0.224 | – | -0.055 | ) | **-3.24** |
| Smoker | -0.016 | ( | -0.143 | – | 0.111 | ) | -0.25 | -0.013 | ( | -0.140 | – | 0.114 | ) | -0.2 | 0.022 | ( | -0.124 | – | 0.167 | ) | 0.29 |
| FH of HD | 0.007 | ( | -0.069 | – | 0.083 | ) | 0.19 | -0.012 | ( | -0.088 | – | 0.064 | ) | -0.31 | -0.026 | ( | -0.108 | – | 0.056 | ) | -0.63 |
| RBC [10 count 10^12^/L increment] | -0.009 | ( | -0.029 | – | 0.011 | ) | -0.9 | -0.010 | ( | -0.030 | – | 0.010 | ) | -0.98 | -0.010 | ( | -0.031 | – | 0.011 | ) | -0.95 |
| WBC [10 count 10^9^/L increment] | 0.000 | ( | -0.001 | – | 0.000 | ) | -1.75 | 0.000 | ( | -0.001 | – | 0.000 | ) | -1.41 | 0.000 | ( | -0.001 | – | 0.000 | ) | -1.67 |
| PLT [1 count 10^9^/L increment] | 0.006 | ( | -0.003 | – | 0.016 | ) | 1.35 | 0.006 | ( | -0.003 | – | 0.015 | ) | 1.25 | 0.006 | ( | -0.003 | – | 0.016 | ) | 1.28 |
| Hemoglobin [1 mg/dL increment] | 0.024 | ( | -0.032 | – | 0.080 | ) | 0.84 | 0.030 | ( | -0.026 | – | 0.087 | ) | 1.05 | 0.032 | ( | -0.028 | – | 0.091 | ) | 1.05 |
| HDL-C [1 mg/dL increment] | -0.001 | ( | -0.004 | – | 0.002 | ) | -0.7 | -0.001 | ( | -0.004 | – | 0.003 | ) | -0.45 | -0.001 | ( | -0.005 | – | 0.002 | ) | -0.69 |
| LDL-C [1 mg/dL increment] | 0.001 | ( | -0.001 | – | 0.002 | ) | 0.69 | 0.001 | ( | -0.001 | – | 0.002 | ) | 0.93 | 0.001 | ( | -0.001 | – | 0.002 | ) | 0.82 |
| Triglycerides [1 mg/dL increment] | 0.000 | ( | -0.001 | – | 0.001 | ) | -0.03 | 0.000 | ( | 0.000 | – | 0.001 | ) | 0.4 | 0.000 | ( | -0.001 | – | 0.001 | ) | 0.33 |
| HbA1c [1 % increment] | 0.153 | ( | 0.049 | – | 0.257 | ) | **2.87** | 0.190 | ( | 0.086 | – | 0.294 | ) | **3.58** | 0.250 | ( | 0.137 | – | 0.364 | ) | **4.32** |
| HOMA-IR [1-point increment] | -0.023 | ( | -0.072 | – | 0.025 | ) | -0.95 | -0.051 | ( | -0.103 | – | 0.001 | ) | -1.94 | -0.077 | ( | -0.135 | – | -0.019 | ) | **-2.62** |
| AST [1 IU/L increment] | -0.004 | ( | -0.012 | – | 0.004 | ) | -1.02 | -0.005 | ( | -0.012 | – | 0.003 | ) | -1.23 | -0.006 | ( | -0.013 | – | 0.002 | ) | -1.41 |
| ALT [1 IU/L increment] | 0.003 | ( | -0.002 | – | 0.009 | ) | 1.25 | 0.004 | ( | -0.002 | – | 0.009 | ) | 1.37 | 0.004 | ( | -0.002 | – | 0.009 | ) | 1.28 |
| γ-GTP [1 IU/L increment] | 0.000 | ( | -0.002 | – | 0.002 | ) | -0.11 | 0.000 | ( | -0.002 | – | 0.001 | ) | -0.32 | 0.000 | ( | -0.002 | – | 0.002 | ) | -0.33 |
| Comorbidity score [1-point increment] | 0.146 | ( | 0.094 | – | 0.198 | ) | **5.52** | 0.128 | ( | 0.076 | – | 0.180 | ) | **4.81** | 0.133 | ( | 0.075 | – | 0.191 | ) | **4.52** |
| Good sleep status | 0.036 | ( | -0.042 | – | 0.115 | ) | 0.91 | 0.035 | ( | -0.044 | – | 0.113 | ) | 0.87 | 0.028 | ( | -0.055 | – | 0.112 | ) | 0.66 |
| Hand grip strength [1 kg increment] | -0.005 | ( | -0.015 | – | 0.006 | ) | -0.83 | -0.004 | ( | -0.015 | – | 0.007 | ) | -0.73 | -0.002 | ( | -0.013 | – | 0.010 | ) | -0.32 |
| Leg power [10 w increment] | 0.004 | ( | 0.002 | – | 0.006 | ) | **3.83** | 0.004 | ( | 0.002 | – | 0.005 | ) | **3.66** | 0.004 | ( | 0.001 | – | 0.006 | ) | **3.3** |
| Seated forward bend [1 cm increment] | 0.001 | ( | -0.005 | – | 0.006 | ) | 0.23 | 0.000 | ( | -0.005 | – | 0.005 | ) | -0.03 | 0.001 | ( | -0.005 | – | 0.006 | ) | 0.23 |
| Step counts [1000 steps/day increment] | -0.003 | ( | -0.015 | – | 0.009 | ) | -0.5 | -0.002 | ( | -0.014 | – | 0.010 | ) | -0.34 | 0.000 | ( | -0.013 | – | 0.013 | ) | 0.03 |
| n-3/n-6 FA ratio [1-point increment] | -0.136 | ( | -0.690 | – | 0.417 | ) | -0.48 | -0.097 | ( | -0.671 | – | 0.478 | ) | -0.33 | -0.125 | ( | -0.730 | – | 0.481 | ) | -0.4 |
| SFA intake [1% energy/day increment] | 0.000 | ( | -0.023 | – | 0.022 | ) | -0.04 | 0.008 | ( | -0.015 | – | 0.031 | ) | 0.69 | 0.001 | ( | -0.023 | – | 0.025 | ) | 0.12 |
| Alcohol consumption [1% energy/day increment] | -0.007 | ( | -0.015 | – | 0.001 | ) | -1.75 | -0.007 | ( | -0.015 | – | 0.001 | ) | -1.77 | -0.008 | ( | -0.017 | – | 0.000 | ) | -1.92 |
| Salt intake [1 g/1,000 kcal/day increment] | 0.039 | ( | 0.005 | – | 0.073 | ) | **2.28** | 0.038 | ( | 0.004 | – | 0.072 | ) | **2.21** | 0.028 | ( | -0.008 | – | 0.064 | ) | 1.54 |
| Sugar intake [1% energy/day increment] | 0.029 | ( | 0.005 | – | 0.053 | ) | **2.4** | 0.035 | ( | 0.011 | – | 0.058 | ) | **2.88** | 0.032 | ( | 0.008 | – | 0.057 | ) | **2.56** |
| Meat intake [10 g/1,000 kcal/day increment] | 0.010 | ( | -0.012 | – | 0.031 | ) | 0.9 | 0.003 | ( | -0.018 | – | 0.024 | ) | 0.29 | -0.002 | ( | -0.024 | – | 0.020 | ) | -0.16 |
| FV intake [10 g/1,000 kcal/day increment] | -0.003 | ( | -0.007 | – | 0.001 | ) | -1.55 | -0.003 | ( | -0.007 | – | 0.001 | ) | -1.59 | -0.005 | ( | -0.009 | – | -0.001 | ) | -2.5 |
| Pulses intake [10 g/1,000 kcal/day increment] | -0.012 | ( | -0.028 | – | 0.003 | ) | -1.53 | -0.012 | ( | -0.027 | – | 0.004 | ) | -1.49 | -0.009 | ( | -0.026 | – | 0.007 | ) | -1.1 |

The values are shown as regression coefficients and 95% confidence intervals for the effect of IMT per unit increment of each covariate. The regression coefficients and 95% confidence intervals are shown in the estimated value corrected by 10 because the estimated value was small. The z scores indicated in bold are statistically significant (≤-1.96 or ≥1.96). Sex, area, and FH of HD were time-stable variables, while the other covariates were time-varying variables. ALT, alanine aminotransferase; AST, aspartate aminotransferase; BCC, biological vascular aging indicator complete case; BMI, body mass index; CC, complete case; FA, fatty acid; FAS, full analysis set; FH, family history; FV, fruits and vegetables; HbA1c, hemoglobin A1C; HD, heart disease; HDL-C, high density lipoprotein cholesterol; HOMA-IR, homeostasis model assessment of insulin resistance; IMT, common carotid intima-media thickness; LDL-C, low density lipoprotein cholesterol; PLT, platelets; RBC, red blood cell count; RC, regression coefficient; SFA, saturated fatty acid; WBC, white blood cell count; γ-GTP, γ-glutamyl transpeptidase

**Supplemental Table 14**. Multivariate panel data analysis for PWV and vascular aging–related covariate trajectory

|  | FAS (*n* = 685) [Measurements = 3,279] | | | | | | | BCC (*n* = 678) [Measurements = 2,936] | | | | | | | CC (*n* = 648) [Measurements = 2,633] | | | | | | |
| --- | --- | --- | --- | --- | --- | --- | --- | --- | --- | --- | --- | --- | --- | --- | --- | --- | --- | --- | --- | --- | --- |
|  | RC | 95% CI | | | | | z score | RC | 95% CI | | | | | z score | RC | 95% CI | | | | | z score |
| Variables | *R^2^* = 0.316 | | | | | | | *R^2^* = 0.313 | | | | | | | *R^2^* = 0.296 | | | | | | |
| Age [1-year increment] | 9.681 | ( | 8.327 | – | 11.034 | ) | **14.02** | 9.991 | ( | 8.579 | – | 11.403 | ) | **13.86** | 9.752 | ( | 8.273 | – | 11.232 | ) | **12.92** |
| Female sex | -54.687 | ( | -103.845 | – | -5.529 | ) | **-2.18** | -56.963 | ( | -108.204 | – | -5.722 | ) | **-2.18** | -59.885 | ( | -112.871 | – | -6.900 | ) | **-2.22** |
| Local area | 39.017 | ( | 7.810 | – | 70.225 | ) | **2.45** | 57.903 | ( | 25.132 | – | 90.674 | ) | **3.46** | 59.559 | ( | 25.835 | – | 93.283 | ) | **3.46** |
| BMI [1 kg/m^2^ increment] | -3.504 | ( | -8.311 | – | 1.303 | ) | -1.43 | -4.010 | ( | -9.057 | – | 1.036 | ) | -1.56 | -4.240 | ( | -9.448 | – | 0.967 | ) | -1.6 |
| Waist/hip ratio [0.1-point increment] | -11.086 | ( | -28.283 | – | 6.111 | ) | -1.26 | -10.012 | ( | -28.016 | – | 7.992 | ) | -1.09 | -8.954 | ( | -27.633 | – | 9.724 | ) | -0.94 |
| Smoker | 18.171 | ( | -6.450 | – | 42.791 | ) | 1.45 | 18.949 | ( | -7.192 | – | 45.091 | ) | 1.42 | 18.220 | ( | -10.298 | – | 46.737 | ) | 1.25 |
| FH of HD | 3.680 | ( | -11.676 | – | 19.037 | ) | 0.47 | -4.647 | ( | -21.737 | – | 12.443 | ) | -0.53 | -2.375 | ( | -20.579 | – | 15.829 | ) | -0.26 |
| RBC [10 count 10^12^/L increment] | 2.353 | ( | -1.714 | – | 6.420 | ) | 1.13 | 1.839 | ( | -2.404 | – | 6.082 | ) | 0.85 | 2.412 | ( | -1.982 | – | 6.806 | ) | 1.08 |
| WBC [10 count 10^9^/L increment] | 0.054 | ( | -0.015 | – | 0.123 | ) | 1.54 | 0.044 | ( | -0.030 | – | 0.117 | ) | 1.16 | 0.048 | ( | -0.028 | – | 0.125 | ) | 1.23 |
| PLT [1 count 10^9^/L increment] | -0.101 | ( | -1.354 | – | 1.152 | ) | -0.16 | 0.360 | ( | -1.605 | – | 2.325 | ) | 0.36 | 0.727 | ( | -1.315 | – | 2.769 | ) | 0.7 |
| Hemoglobin [1 mg/dL increment] | 8.061 | ( | -3.631 | – | 19.753 | ) | 1.35 | 9.144 | ( | -3.155 | – | 21.443 | ) | 1.46 | 6.236 | ( | -6.564 | – | 19.035 | ) | 0.95 |
| HDL-C [1 mg/dL increment] | 0.062 | ( | -0.593 | – | 0.717 | ) | 0.19 | 0.077 | ( | -0.605 | – | 0.758 | ) | 0.22 | 0.028 | ( | -0.681 | – | 0.737 | ) | 0.08 |
| LDL-C [1 mg/dL increment] | -0.345 | ( | -0.641 | – | -0.049 | ) | **-2.28** | -0.372 | ( | -0.684 | – | -0.060 | ) | **-2.34** | -0.380 | ( | -0.712 | – | -0.049 | ) | **-2.25** |
| Triglycerides [1 mg/dL increment] | 0.088 | ( | -0.046 | – | 0.223 | ) | 1.29 | 0.101 | ( | -0.037 | – | 0.238 | ) | 1.44 | 0.142 | ( | -0.030 | – | 0.314 | ) | 1.62 |
| HbA1c [1 % increment] | 4.353 | ( | -17.120 | – | 25.826 | ) | 0.4 | 7.000 | ( | -15.742 | – | 29.743 | ) | 0.6 | 6.275 | ( | -18.367 | – | 30.917 | ) | 0.5 |
| HOMA-IR [1-point increment] | 11.979 | ( | 2.342 | – | 21.615 | ) | **2.44** | 9.679 | ( | -2.125 | – | 21.482 | ) | 1.61 | 12.790 | ( | 0.301 | – | 25.881 | ) | **1.98** |
| AST [1 IU/L increment] | 2.831 | ( | 1.203 | – | 4.460 | ) | **3.41** | 3.112 | ( | 1.411 | – | 4.813 | ) | **3.59** | 3.382 | ( | 1.633 | – | 5.131 | ) | **3.79** |
| ALT [1 IU/L increment] | -1.908 | ( | -3.054 | – | -0.762 | ) | **-3.26** | -1.919 | ( | -3.127 | – | -0.710 | ) | **-3.11** | -2.200 | ( | -3.438 | – | -0.961 | ) | **-3.48** |
| γ-GTP [1 IU/L increment] | 0.150 | ( | -0.214 | – | 0.514 | ) | 0.81 | 0.075 | ( | -0.298 | – | 0.448 | ) | 0.39 | 0.077 | ( | -0.316 | – | 0.470 | ) | 0.38 |
| Comorbidity score [1-point increment] | 26.552 | ( | 15.727 | – | 37.377 | ) | **4.81** | 28.846 | ( | 17.113 | – | 40.580 | ) | **4.82** | 25.413 | ( | 12.517 | – | 38.310 | ) | **3.86** |
| Good sleep status | 1.386 | ( | -15.615 | – | 18.387 | ) | 0.16 | 1.975 | ( | -15.929 | – | 19.879 | ) | 0.22 | 0.328 | ( | -18.590 | – | 19.246 | ) | 0.03 |
| Hand grip strength [1 kg increment] | 1.405 | ( | -0.828 | – | 3.638 | ) | 1.23 | 0.919 | ( | -1.407 | – | 3.245 | ) | 0.77 | 0.794 | ( | -1.618 | – | 3.205 | ) | 0.65 |
| Leg power [10 w increment] | 0.115 | ( | -0.302 | – | 0.532 | ) | 0.54 | 0.202 | ( | -0.224 | – | 0.628 | ) | 0.93 | 0.290 | ( | -0.173 | – | 0.752 | ) | 1.23 |
| Seated forward bend [1 cm increment] | -1.791 | ( | -2.840 | – | -0.742 | ) | **-3.35** | -1.770 | ( | -2.872 | – | -0.668 | ) | **-3.15** | -1.840 | ( | -2.994 | – | -0.687 | ) | **-3.13** |
| Step counts [1000 steps/day increment] | -1.822 | ( | -4.379 | – | 0.735 | ) | -1.4 | -1.853 | ( | -4.522 | – | 0.815 | ) | -1.36 | -1.261 | ( | -4.022 | – | 1.500 | ) | -0.9 |
| n-3/n-6 FA ratio [1-point increment] | 41.337 | ( | -84.527 | – | 167.201 | ) | 0.64 | -16.637 | ( | -149.291 | – | 116.018 | ) | -0.25 | 8.759 | ( | -130.318 | – | 147.836 | ) | 0.12 |
| SFA intake [1 % energy/day increment] | 3.059 | ( | -1.890 | – | 8.008 | ) | 1.21 | 3.156 | ( | -2.023 | – | 8.335 | ) | 1.19 | 2.021 | ( | -3.449 | – | 7.490 | ) | 0.72 |
| Alcohol consumption [1 % energy/day increment] | 1.092 | ( | -0.602 | – | 2.786 | ) | 1.26 | 1.407 | ( | -0.369 | – | 3.184 | ) | 1.55 | 1.455 | ( | -0.388 | – | 3.298 | ) | 1.55 |
| Salt intake [1 g/1,000 kcal/day increment] | 0.344 | ( | -7.027 | – | 7.715 | ) | 0.09 | 3.952 | ( | -3.782 | – | 11.686 | ) | 1 | 5.677 | ( | -2.510 | – | 13.864 | ) | 1.36 |
| Sugar intake [1 % energy/day increment] | 0.497 | ( | -4.570 | – | 5.564 | ) | 0.19 | -0.346 | ( | -5.683 | – | 4.991 | ) | -0.13 | 0.047 | ( | -5.537 | – | 5.630 | ) | 0.02 |
| Meat intake [10 g/1,000 kcal/day increment] | 2.694 | ( | -1.949 | – | 7.338 | ) | 1.14 | 2.899 | ( | -1.947 | – | 7.744 | ) | 1.17 | 2.681 | ( | -2.410 | – | 7.773 | ) | 1.03 |
| FV intake [10 g/1,000 kcal/day increment] | 0.577 | ( | -0.269 | – | 1.424 | ) | 1.34 | 0.218 | ( | -0.669 | – | 1.106 | ) | 0.48 | 0.273 | ( | -0.665 | – | 1.210 | ) | 0.57 |
| Pulses intake [10 g/1,000 kcal/day increment] | 0.482 | ( | -2.921 | – | 3.885 | ) | 0.28 | 0.120 | ( | -3.453 | – | 3.692 | ) | 0.07 | 1.087 | ( | -2.704 | – | 4.879 | ) | 0.56 |

The values are shown as regression coefficients and 95% confidence intervals for the effect of PWV per unit increment of each covariate. The z scores indicated in bold are statistically significant (≤-1.96 or ≥1.96). Sex, area, and FH of HD were time-stable variables, while the other covariates were time-varying variables. ALT, alanine aminotransferase; AST, aspartate aminotransferase; BCC, biological vascular aging indicator complete case; BMI, body mass index; CC, complete case; FA, fatty acid; FAS, full analysis set; FH, family history; FV, fruits and vegetables; HbA1c, hemoglobin A1C; HD, heart disease; HDL-C, high density lipoprotein cholesterol; HOMA-IR, homeostasis model assessment of insulin resistance; LDL-C, low density lipoprotein cholesterol; PLT, platelets; PWV, carotid-femoral pulse wave velocity; RBC, red blood cell count; RC, regression coefficient; SFA, saturated fatty acid; WBC, white blood cell count; γ-GTP, γ-glutamyl transpeptidase

**Supplemental Table 15**. Multivariate panel data analysis for VI and vascular aging–related covariate trajectory

|  | FAS (*n* = 678) [Measurements = 2,943] | | | | | | | BCC (*n* = 678) [Measurements = 2,936] | | | | | | | CC (*n* = 648) [Measurements = 2,633] | | | | | | |
| --- | --- | --- | --- | --- | --- | --- | --- | --- | --- | --- | --- | --- | --- | --- | --- | --- | --- | --- | --- | --- | --- |
|  | RC | 95% CI | | | | | z score | RC | 95% CI | | | | | z score | RC | 95% CI | | | | | z score |
| Variables | *R^2^* = 0.417 | | | | | | | *R^2^* = 0.416 | | | | | | | *R^2^* = 0.394 | | | | | | |
| Age [1-year increment] | 0.346 | ( | 0.312 | – | 0.380 | ) | **19.78** | 0.346 | ( | 0.311 | – | 0.380 | ) | **19.78** | 0.336 | ( | 0.300 | – | 0.371 | ) | **18.32** |
| Female sex | -1.383 | ( | -2.626 | – | -0.139 | ) | **-2.18** | -1.384 | ( | -2.629 | – | -0.139 | ) | **-2.18** | -1.385 | ( | -2.674 | – | -0.097 | ) | **-2.11** |
| Local area | 1.274 | ( | 0.470 | – | 2.079 | ) | **3.1** | 1.304 | ( | 0.499 | – | 2.110 | ) | **3.17** | 1.317 | ( | 0.488 | – | 2.146 | ) | **3.11** |
| BMI [1 kg/m^2^ increment] | -0.003 | ( | -0.126 | – | 0.120 | ) | -0.05 | -0.011 | ( | -0.134 | – | 0.112 | ) | -0.17 | -0.013 | ( | -0.140 | – | 0.114 | ) | -0.2 |
| Waist/hip ratio [0.1-point increment] | -0.482 | ( | -0.908 | – | -0.055 | ) | **-2.21** | -0.446 | ( | -0.872 | – | -0.020 | ) | **-2.05** | -0.417 | ( | -0.858 | – | 0.025 | ) | -1.85 |
| Smoker | 0.437 | ( | -0.197 | – | 1.070 | ) | 1.35 | 0.436 | ( | -0.197 | – | 1.069 | ) | 1.35 | 0.462 | ( | -0.237 | – | 1.161 | ) | 1.3 |
| FH of HD | -0.149 | ( | -0.553 | – | 0.255 | ) | -0.72 | -0.140 | ( | -0.543 | – | 0.264 | ) | -0.68 | -0.109 | ( | -0.539 | – | 0.321 | ) | -0.5 |
| RBC [10 count 10^12^/L increment] | 0.035 | ( | -0.067 | – | 0.137 | ) | 0.67 | 0.030 | ( | -0.071 | – | 0.132 | ) | 0.59 | 0.044 | ( | -0.061 | – | 0.150 | ) | 0.83 |
| WBC [10 count 10^9^/L increment] | 0.001 | ( | -0.001 | – | 0.002 | ) | 0.69 | 0.000 | ( | -0.001 | – | 0.002 | ) | 0.54 | 0.000 | ( | -0.001 | – | 0.002 | ) | 0.54 |
| PLT [1 count 10^9^/L increment] | 0.011 | ( | -0.036 | – | 0.059 | ) | 0.48 | 0.014 | ( | -0.033 | – | 0.061 | ) | 0.58 | 0.024 | ( | -0.025 | – | 0.073 | ) | 0.95 |
| Hemoglobin [1 mg/dL increment] | 0.202 | ( | -0.091 | – | 0.496 | ) | 1.35 | 0.213 | ( | -0.080 | – | 0.506 | ) | 1.43 | 0.148 | ( | -0.157 | – | 0.452 | ) | 0.95 |
| HDL-C [1 mg/dL increment] | 0.000 | ( | -0.016 | – | 0.017 | ) | 0.05 | 0.000 | ( | -0.016 | – | 0.017 | ) | 0.03 | -0.002 | ( | -0.019 | – | 0.015 | ) | -0.23 |
| LDL-C [1 mg/dL increment] | -0.007 | ( | -0.014 | – | 0.001 | ) | -1.74 | -0.006 | ( | -0.014 | – | 0.001 | ) | -1.71 | -0.007 | ( | -0.014 | – | 0.001 | ) | -1.63 |
| Triglycerides [1 mg/dL increment] | 0.002 | ( | -0.001 | – | 0.006 | ) | 1.42 | 0.002 | ( | -0.001 | – | 0.006 | ) | 1.46 | 0.003 | ( | -0.001 | – | 0.007 | ) | 1.62 |
| HbA1c [1 % increment] | 0.413 | ( | -0.129 | – | 0.954 | ) | 1.49 | 0.416 | ( | -0.124 | – | 0.957 | ) | 1.51 | 0.464 | ( | -0.123 | – | 1.050 | ) | 1.55 |
| HOMA-IR [1-point increment] | 0.143 | ( | -0.135 | – | 0.421 | ) | 1.01 | 0.136 | ( | -0.140 | – | 0.413 | ) | 0.97 | 0.175 | ( | -0.132 | – | 0.482 | ) | 1.12 |
| AST [1 IU/L increment] | 0.063 | ( | 0.022 | – | 0.103 | ) | **3.06** | 0.063 | ( | 0.023 | – | 0.103 | ) | **3.09** | 0.068 | ( | 0.027 | – | 0.109 | ) | **3.25** |
| ALT [1 IU/L increment] | -0.036 | ( | -0.064 | – | -0.007 | ) | **-2.46** | -0.036 | ( | -0.065 | – | -0.008 | ) | **-2.49** | -0.043 | ( | -0.072 | – | -0.014 | ) | **-2.89** |
| γ-GTP [1 IU/L increment] | 0.001 | ( | -0.008 | – | 0.010 | ) | 0.13 | 0.000 | ( | -0.008 | – | 0.009 | ) | 0.1 | 0.001 | ( | -0.009 | – | 0.010 | ) | 0.14 |
| Comorbidity score [1-point increment] | 0.801 | ( | 0.524 | – | 1.078 | ) | **5.66** | 0.806 | ( | 0.529 | – | 1.082 | ) | **5.71** | 0.728 | ( | 0.425 | – | 1.032 | ) | **4.7** |
| Good sleep status | 0.095 | ( | -0.326 | – | 0.516 | ) | 0.44 | 0.104 | ( | -0.317 | – | 0.524 | ) | 0.48 | 0.065 | ( | -0.380 | – | 0.509 | ) | 0.28 |
| Hand grip strength [1 kg increment] | 0.015 | ( | -0.041 | – | 0.070 | ) | 0.51 | 0.013 | ( | -0.042 | – | 0.069 | ) | 0.47 | 0.013 | ( | -0.045 | – | 0.071 | ) | 0.45 |
| Leg power [10 w increment] | 0.009 | ( | -0.001 | – | 0.019 | ) | 1.74 | 0.010 | ( | -0.001 | – | 0.020 | ) | 1.86 | 0.011 | ( | 0.000 | – | 0.022 | ) | **2.03** |
| Seated forward bend [1 cm increment] | -0.040 | ( | -0.066 | – | -0.014 | ) | **-2.99** | -0.040 | ( | -0.067 | – | -0.014 | ) | **-3.01** | -0.042 | ( | -0.069 | – | -0.014 | ) | **-2.95** |
| Step counts [1000 steps/day increment] | -0.041 | ( | -0.104 | – | 0.023 | ) | -1.26 | -0.039 | ( | -0.103 | – | 0.024 | ) | -1.22 | -0.022 | ( | -0.087 | – | 0.044 | ) | -0.65 |
| n-3/n-6 FA ratio [1-point increment] | -0.534 | ( | -3.644 | – | 2.575 | ) | -0.34 | -0.563 | ( | -3.665 | – | 2.538 | ) | -0.36 | 0.040 | ( | -3.208 | – | 3.289 | ) | 0.02 |
| SFA intake [1 % energy/day increment] | 0.077 | ( | -0.045 | – | 0.198 | ) | 1.24 | 0.085 | ( | -0.037 | – | 0.206 | ) | 1.37 | 0.050 | ( | -0.078 | – | 0.178 | ) | 0.76 |
| Alcohol consumption [1 % energy/day increment] | 0.020 | ( | -0.023 | – | 0.062 | ) | 0.91 | 0.018 | ( | -0.024 | – | 0.060 | ) | 0.83 | 0.017 | ( | -0.027 | – | 0.061 | ) | 0.76 |
| Salt intake [1 g/1,000 kcal/day increment] | 0.037 | ( | -0.144 | – | 0.219 | ) | 0.4 | 0.034 | ( | -0.148 | – | 0.215 | ) | 0.36 | 0.087 | ( | -0.104 | – | 0.279 | ) | 0.89 |
| Sugar intake [1% energy/day increment] | 0.048 | ( | -0.078 | – | 0.174 | ) | 0.74 | 0.056 | ( | -0.070 | – | 0.181 | ) | 0.87 | 0.041 | ( | -0.090 | – | 0.172 | ) | 0.61 |
| Meat intake [10 g/1,000 kcal/day increment] | 0.070 | ( | -0.044 | – | 0.184 | ) | 1.2 | 0.067 | ( | -0.047 | – | 0.180 | ) | 1.15 | 0.057 | ( | -0.062 | – | 0.176 | ) | 0.94 |
| FV intake [10 g/1,000 kcal/day increment] | 0.000 | ( | -0.021 | – | 0.021 | ) | -0.01 | -0.001 | ( | -0.022 | – | 0.019 | ) | -0.13 | -0.003 | ( | -0.025 | – | 0.019 | ) | -0.3 |
| Pulses intake [10 g/1,000 kcal/day increment] | -0.012 | ( | -0.096 | – | 0.072 | ) | -0.28 | -0.011 | ( | -0.095 | – | 0.073 | ) | -0.26 | 0.015 | ( | -0.073 | – | 0.104 | ) | 0.34 |

The values are shown as regression coefficients and 95% confidence intervals for the effect of VI per unit increment of each covariate. The z scores indicated in bold are statistically significant (≤-1.96 or ≥1.96). Sex, area, and FH of HD were time-stable variables, while the other covariates were time-varying variables. ALT, alanine aminotransferase; AST, aspartate aminotransferase; BCC, biological vascular aging indicator complete case; BMI, body mass index; CC, complete case; FA, fatty acid; FAS, full analysis set; FH, family history; FV, fruits and vegetables; HbA1c, hemoglobin A1C; HD, heart disease; HDL-C, high density lipoprotein cholesterol; HOMA-IR, homeostasis model assessment of insulin resistance; LDL-C, low density lipoprotein cholesterol; PLT, platelets; RBC, red blood cell count; RC, regression coefficient; SFA, saturated fatty acid; VI, vascular aging index; WBC, white blood cell count; γ-GTP, γ-glutamyl transpeptidase


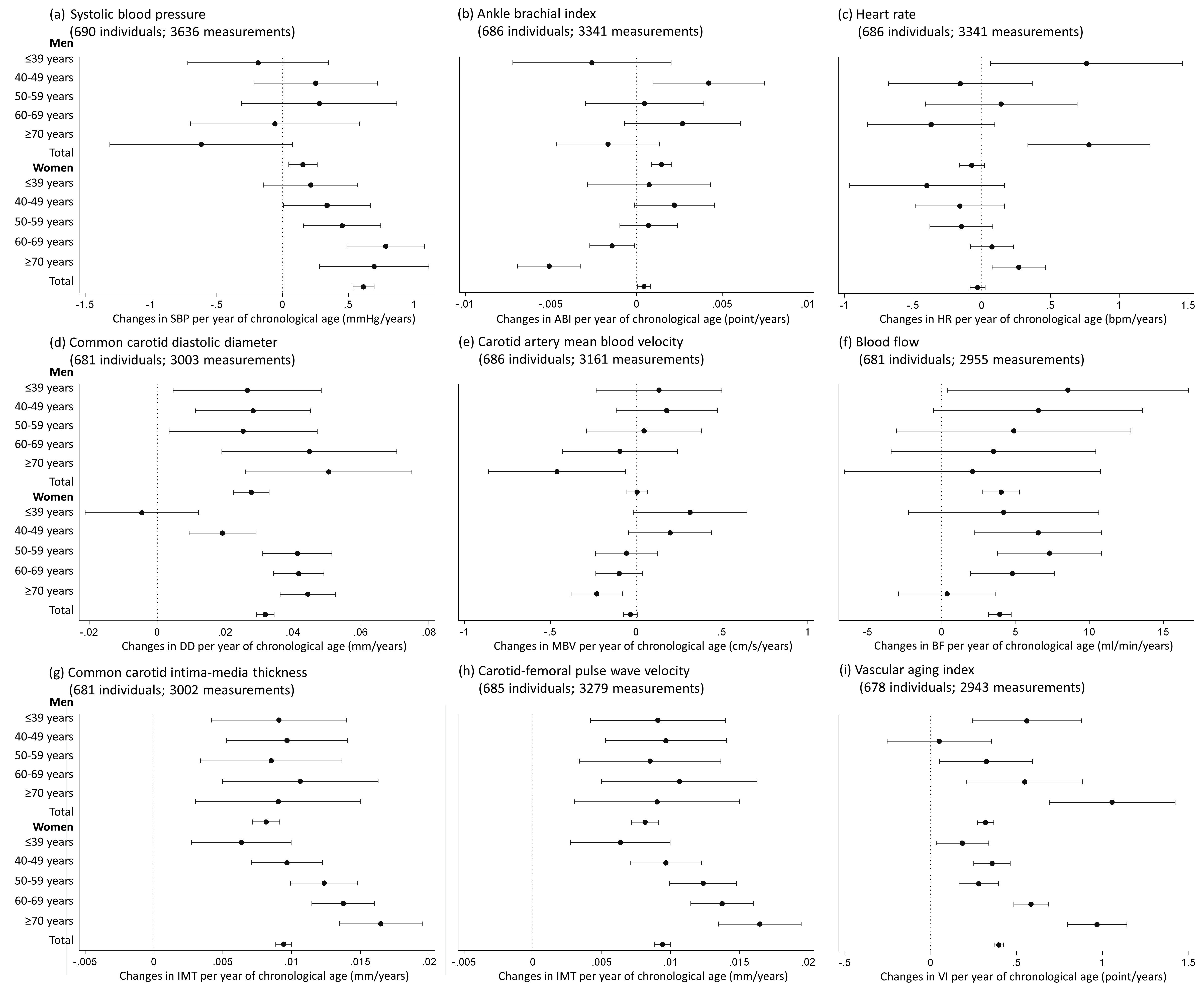


**Supplemental Figure 1**. Effect of BVAIs per year of chronological age by age and sex-stratified model

The values are shown as regression coefficients and 95% confidence intervals.

**
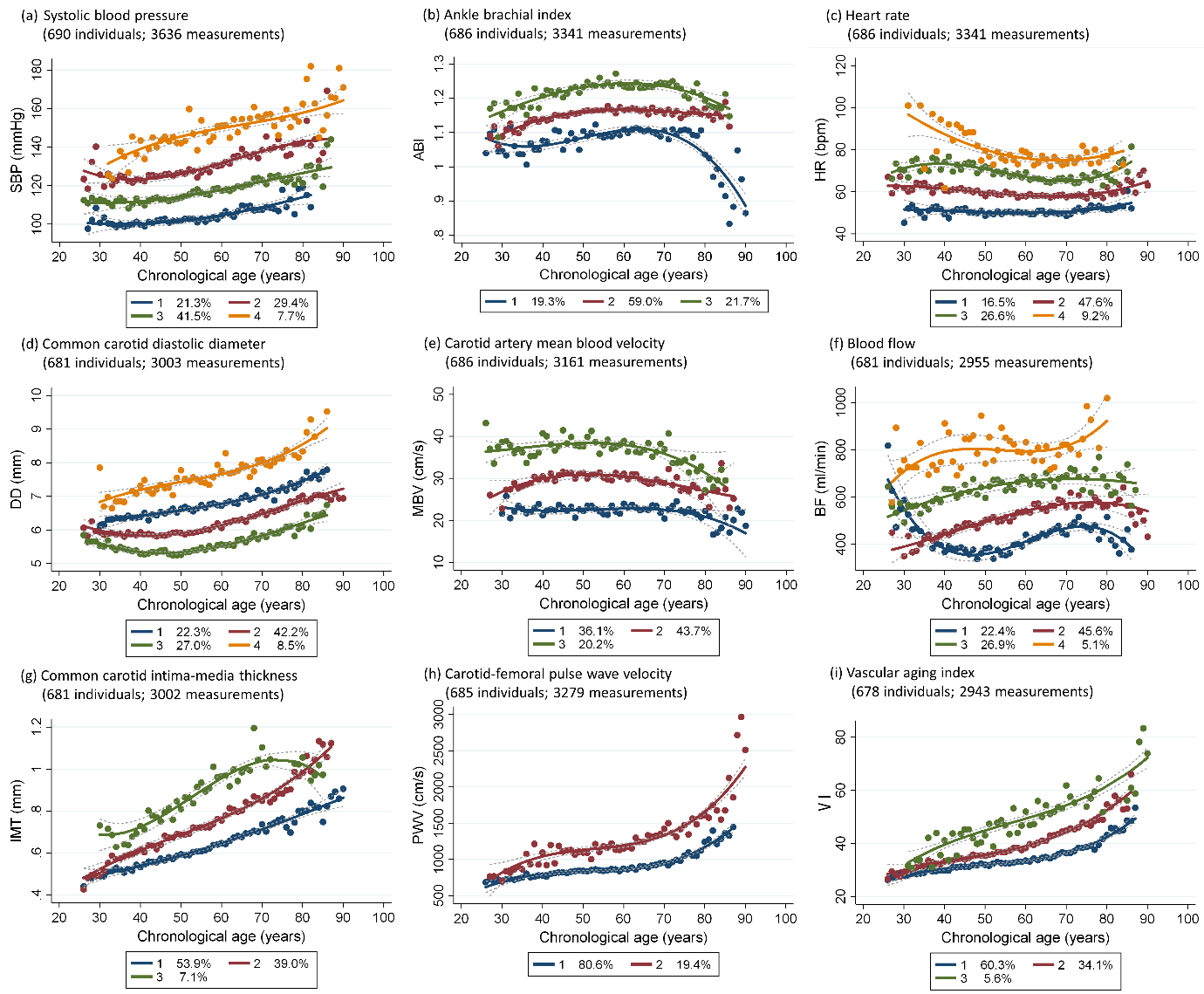
**

**Supplemental Figure 2**. Trajectory groups by trajectory modeling for nine BVAIs. Solid lines represent the mean value of each trajectory group; dashed lines represent 95% confidence intervals. The latent class growth models identified 2 to 4 distinct trajectory groups according to the BVAIs, in participants aged 26–90 years, using the maximum likelihood method.
